# Source-Specific Photobiomodulation Regulates Mitochondrial Bioenergetics, Redox Signaling, and Functional Outputs in C2C12 Myoblasts Across Replicative Aging

**DOI:** 10.3390/ijms27072999

**Published:** 2026-03-25

**Authors:** Ana Elena Aviña, Nguyen Le Thanh Hang, Che-Yi Chang, Yi-Fan Chen, Yun Yen, Xavier Pei-Chun Wong, Aline Yen Ling Wang, Cheng-Jen Chang, Tzu-Sen Yang

**Affiliations:** 1 International Ph.D. Program in Medicine, College of Medicine, Taipei Medical University, Taipei 110, Taiwan; d142111012@tmu.edu.tw; 2 Graduate Institute of Biomedical Optomechatronics, College of Biomedical Engineering, Taipei Medical University, New Taipei City 235, Taiwan; d845111005@tmu.edu.tw (N.L.T.H.); pcwong0424@tmu.edu.tw (X.P.-C.W.); 3 Department of Plastic Surgery, Taipei Medical University Hospital, Taipei Medical University, Taipei 110, Taiwan; 4 Center for Vascularized Composite Allotransplantation, Chang Gung Memorial Hospital, Taoyuan 333, Taiwan; 5 Department of Otolaryngology, National Taiwan University Hospital, Taipei 100, Taiwan; 6 Graduate Institute of Biomedical Materials and Tissue Engineering, Taipei Medical University, Taipei 110, Taiwan; orzdinoqoo1@gmail.com; 7 International Master/Ph.D. Program for Translation Science, College of Medical Science and Technology, Taipei Medical University, New Taipei City 235, Taiwan; evan.yifan@tmu.edu.tw; 8 TMU Research Center of Cancer Translational Medicine, Taipei Medical University, Taipei 110, Taiwan; yyen@tmu.edu.tw; 9 College of Biomedical Engineering, Taipei Medical University, New Taipei City 235, Taiwan; 10 Department of Surgery, School of Medicine, College of Medicine, Taipei Medical University, Taipei 110, Taiwan; 11 International Ph.D. Program in Biomedical Engineering, Taipei Medical University, New Taipei City 235, Taiwan; 12 School of Dental Technology, Taipei Medical University, Taipei 110, Taiwan; 13 Research Center of Biomedical Devices, Taipei Medical University, Taipei 110, Taiwan

**Keywords:** photobiomodulation, muscle, aging, C2C12 myoblasts, mitochondria, reactive oxygen species, extracellular vesicles, bioenergetics, NIR, LED

## Abstract

Age-related muscle decline is associated with impaired mitochondrial bioenergetics, altered redox signaling, and reduced myogenic capacity, yet how photobiomodulation (PBM) source characteristics shape these processes under replicative aging remains unclear. Here, we investigated source-specific PBM responses in C2C12 myoblasts using a 660 nm light-emitting diode (LED) and an 830 nm near-infrared (NIR) laser across fluence ranges and replicative stages. Single-cell screening performed at passage 25 identified 5 J/cm^2^ as the optimal fluence for both sources, producing biphasic increases in mitochondrial membrane potential and ROS. Population-level assays in young (≤5 passages) and old (≥30 passages) cells revealed divergent downstream outcomes. LED irradiation elicited stronger metabolic activation and ATP production, particularly in aged cells, whereas NIR irradiation robustly enhanced myogenic fusion in both age groups and partially rescued differentiation deficits in aged myoblasts. Bulk ROS increased significantly after PBM independent of source, while extracellular vesicle release displayed age-dependent source sensitivity, with NIR favoring canonical small EV populations in young cells and LED inducing greater particle release in aged cells. Together, these findings demonstrate that PBM engages conserved mitochondrial signaling while source-specific delivery and wavelength differentially direct metabolic, paracrine, and myogenic outputs under replicative aging conditions.

## 1. Introduction

Photobiomodulation (PBM) is a form of light therapy that employs non-ionizing irradiation using a variety of light sources, including lasers, light-emitting diodes (LEDs), and broadband light, typically within the visible to near-infrared spectrum. PBM is a nonthermal process that triggers photophysical and photochemical interactions with endogenous chromophores, leading to biological responses such as enhanced tissue repair, immunomodulation, and improved cellular function [1,2]. Beyond its therapeutic scope, PBM has been increasingly explored for regenerative medicine applications, where it shows potential to enhance healing and modulate cellular communication.

Mitochondria, particularly cytochrome c oxidase (CcO) in complex IV of the electron transport chain, are widely recognized as key photoacceptors involved in photobiomodulation responses. Upon light absorption, CcO activity may increase, leading to enhanced electron transport, elevation of the mitochondrial membrane potential (ΔΨm)**,** and increased ATP production [3,4,5,6]. In addition, controlled production of reactive oxygen species (ROS) can act as redox signals that regulate cellular adaptation, signaling pathways, and tissue repair processes [7,8,9,10,11,12]. Together, these mechanisms highlight the interplay between mitochondrial bioenergetics, ΔΨm, and ROS as a central component underlying PBM-induced cellular responses.

Because skeletal muscle is highly reliant on mitochondrial capacity for function and repair, PBM has been investigated for its ability to improve exercise performance, accelerate recovery, and mitigate fatigue [13,14]. Nevertheless, interpretability remains challenging: biological outcomes vary across studies depending on wavelength, fluence, and source characteristics, and the biphasic dose–response phenomenon is well established in PBM [15,16]. Few studies have systematically linked mitochondrial responses to functional outputs such as migration, differentiation, or extracellular vesicle (EV) release within a single experimental platform [17,18,19].

This gap is particularly relevant to conditions of age-related muscle loss and weakness (such as sarcopenia) [20,21] in which mitochondrial bioenergetic dysfunction plays a central role. Impaired oxidative phosphorylation, reduced ATP production, and altered mitochondrial dynamics converge with defective myogenic differentiation and disrupted redox signaling, collectively driving the progressive decline of muscle mass and function. Beyond energy failure, mitochondria act as signaling hubs that regulate reactive oxygen species, calcium handling, and apoptotic pathways, meaning their dysfunction propagates systemic stress responses that accelerate tissue frailty. Understanding and targeting these interconnected processes is therefore critical for developing strategies to mitigate muscle aging and restore regenerative potential [21,22,23,24].

Accordingly, we selected two commonly used but physically distinct PBM sources: a red LED at 660 nm and a near-infrared (NIR) laser at 830 nm. These wavelengths were chosen not only because both fall within the “optical window” for tissue penetration, but also because their biological actions are complementary. Red light at 660 nm has a shallower penetration profile, favoring safe, broad-coverage stimulation of superficial or preventive targets, while 830 nm penetrates deeper and is better suited for modulating mitochondrial function in thicker tissue contexts [25,26,27,28]. The use of an LED at 660 nm represents a practical, safer, and more accessible source, while the coherent 830 nm laser provides higher photon density and depth, enabling a side-by-side comparison of how light source properties influence mitochondrial dynamics in the same cellular model [29,30].

The C2C12 myoblast line offers a robust system to explore these questions because it retains strong mitochondrial content, differentiates into multinucleated myotubes, and reproduces key features of muscle physiology [31,32]. Importantly, its well-documented passage-dependent changes allow comparisons between early-passage “young” cells, which maintain proliferative and differentiation capacity, and late-passage “old” cells, which accumulate mitochondrial and functional deficits [33,34]. This makes C2C12 particularly suited to mechanistic interrogation of mitochondrial function under PBM, enabling quantitative single-cell readouts of ΔΨm and ROS, alongside functional assays of migration, differentiation, and EV release.

Here, the objective of this study was to determine whether photobiomodulation (PBM) delivered using a 660 nm LED or an 830 nm near-infrared (NIR) laser differentially affects mitochondrial function in C2C12 myoblasts in a fluence-dependent manner. We hypothesized that PBM delivered by these two light sources would produce source-dependent mitochondrial responses that translate into distinct functional outcomes, including changes in metabolic activity, extracellular vesicle release, migration, and myogenic differentiation under replicative aging conditions. To test this hypothesis, we combined image-based single-cell measurements of mitochondrial activity with population-level functional assays in early- and late-passage myoblasts, enabling evaluation of the relationship between mitochondrial bioenergetics, redox signaling, and cellular behavior following PBM.

## 2. Results

### 2.1. Effects of PBM on Mitochondrial Membrane Potential and ROS at the Single-Cell Level

**Figure 1:**
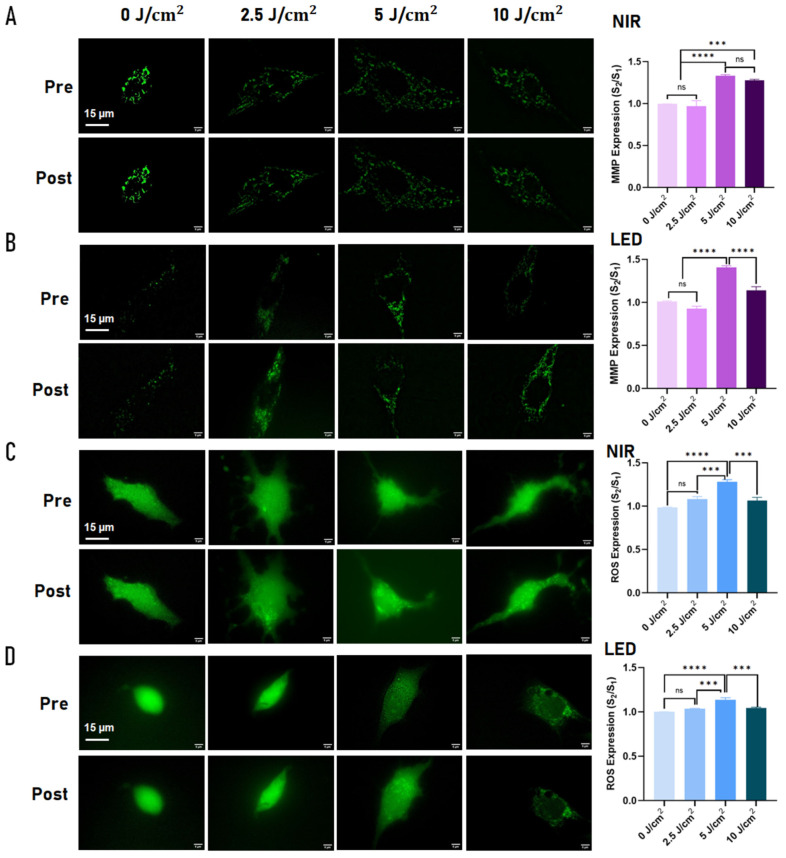
Single-cell analysis of mitochondrial membrane potential (ΔΨm, MMP) and reactive oxygen species (ROS) responses to photobiomodulation (PBM) in C2C12 myoblasts (P25). Each cell was imaged before (Pre) and after (Post) irradiation in the same microchamber, and fluorescence changes were quantified as the normalized intensity ratio (post/pre; S_2_/S_1_). (**A**) Rhodamine 123 + 830 nm NIR. (**B**) Rhodamine 123 + 660 nm LED. (**C**) H_2_DCFDA + NIR. (**D**) H_2_DCFDA + LED. Bar graphs show normalized fluorescence ratios; color intensity reflects increasing PBM fluence (0–10 J/cm^2^). Five cells per experiment were analyzed across three independent experiments (*n* = 15 cells per condition). Data are mean ± SEM; one-way ANOVA with Tukey post hoc test. ns, not significant; *** *p* < 0.001; **** *p* < 0.0001. Scale bar: 15 µm.

To assess acute, source-specific mitochondrial and redox responses to photobiomodulation (PBM), single-cell measurements of mitochondrial membrane potential (ΔΨm, reported as MMP) and intracellular reactive oxygen species (ROS) were performed in C2C12 myoblasts at intermediate passage (P. 25) as an initial dose–response screen (Figure 1). ΔΨm and ROS were quantified using Rhodamine 123 (Rh123) and H_2_DCFDA staining, respectively.

For MMP (ΔΨm), both 830 nm NIR laser and 660 nm LED irradiation produced significant, fluence-dependent increases in Rh123 fluorescence relative to baseline. The largest increase was observed at 5 J/cm^2^, which was significantly higher than untreated controls and the 2.5 J/cm^2^ condition (*p* < 0.0001), while 10 J/cm^2^ did not further augment the response, consistent with a plateau in mitochondrial polarization (Figure 1A,B).

ROS levels also increased following PBM. For both light sources, 5 J/cm^2^ elicited the most consistent elevation in H_2_DCFDA signal relative to baseline, whereas 2.5 J/cm^2^ did not differ from control conditions and 10 J/cm^2^ did not produce an additional significant increase compared with controls in this single-cell readout (Figure 1C,D).

Together, these single-cell data identified 5 J/cm^2^ as a leading candidate dose for downstream testing (Supplementary Table S2). To verify whether this apparent plateau extended to population-level outcomes and to better resolve potential biphasic behavior, a broader fluence range was subsequently evaluated in bulk cultures using CCK-8 viability assays (2.5, 5, 10, and 15 J/cm^2^), and the final working dose was selected based on the combined single-cell and bulk dose–response results.

### 2.2. Effects of PBM on Metabolic Activity, ATP Production, and ROS Signaling in Young and Replicatively Aged C2C12 Cells

**Figure 2:**
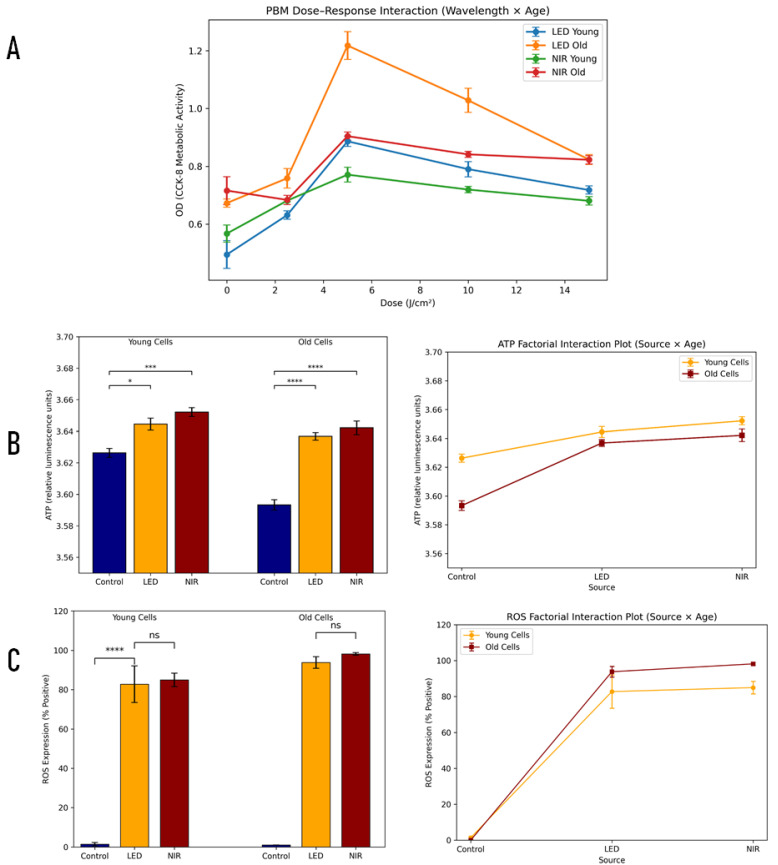
Dose-, wavelength-, and age-dependent metabolic and redox responses to photobiomodulation (PBM) in C2C12 cells. Metabolic activity was assessed using the CCK-8 assay following PBM delivered by 660 nm LED or 830 nm NIR across fluences of 0–15 J/cm^2^ in young (≤5 passages) and old (≥30 passages) cells. (**A**) Consolidated factorial analysis of photobiomodulation responses across wavelength, dose, and age-status. OD values in young and old C2C12 cells exposed to LED or NIR irradiation across increasing fluences (0, 2.5, 5, 10, and 15 J/cm^2^). Data are shown as mean ± SEM (n = 9 per condition). The integrated interaction plot illustrates the combined effects of wavelength, dose, and cellular age-status. Statistical analysis was performed using three-way ANOVA followed by Sidak-adjusted multiple comparisons. (**B**) ATP production measured after PBM at 5 J/cm^2^ for LED and NIR relative to non-irradiated controls. (**C**) Intracellular ROS positivity measured under the same conditions. Bars and points represent mean ± SEM from three independent experiments. Statistical comparisons were performed using two-way ANOVA (source × age) for ATP and ROS, followed by Tukey’s post hoc test. ns, not significant; * *p* < 0.05; *** *p* < 0.001; **** *p* < 0.0001. Color coding differs by panel: in (**A**), blue, LED young; orange, LED old; green, NIR young; red, NIR old. In ((**B**,**C**), left), blue, control; orange, LED; red, NIR (shown separately for young and old cells). In ((**B**,**C**), right), orange, young cells; red, old cells.

To characterize population-level responses to photobiomodulation (PBM) and guide fluence selection for downstream functional assays, metabolic activity was evaluated in young (≤5 passages) and replicatively aged (≥30 passages) C2C12 cells following irradiation with either 660 nm LED or 830 nm NIR across a range of fluences (Figure 2A). Replicative aging of late-passage cultures was qualitatively confirmed by increased senescence-associated β-galactosidase staining compared with early-passage cells (Supplementary Figure S4).

Based on the concordant identification of 5 J/cm^2^ as the optimal fluence in both single-cell mitochondrial assays (Figure 1) and bulk metabolic screening, this dose was selected for subsequent mechanistic and functional analyses like ATP and ROS.

#### 2.2.1. PBM Modulates C2C12 Metabolic Activity in a Dose-, Wavelength-, and Age-Dependent Manner

Cell metabolic activity was assessed using the CCK-8 assay following photobiomodulation with either 660 nm LED or 830 nm NIR irradiation across increasing fluences (0–15 J/cm^2^) in young (≤5 passages) and old (≥30 passages) C2C12 cells (Figure 2A).

To account for the factorial design of the experiment, a three-way ANOVA (Dose × Wavelength × Age) was performed. This analysis revealed highly significant main effects of dose (F_4,160_ ≈ 848.5, *p* < 0.0001), wavelength (F_1,160_ ≈ 252.2, *p* < 0.0001), and cellular age-status (F_1,160_ ≈ 1466.8, *p* < 0.0001), indicating that metabolic activity varied significantly with PBM fluence, irradiation source, and replicative aging. Significant two-way interactions were also detected between dose and wavelength (F_4,160_ ≈ 145.4, *p* < 0.0001), dose and age (F_4,160_ ≈ 49.4, *p* < 0.0001), and wavelength and age (F_1,160_ ≈ 117.5, *p* < 0.0001). Importantly, the three-way interaction (Dose × Wavelength × Age) was also significant (F_4,160_ ≈ 26.0, *p* < 0.0001), indicating that the dose–response pattern differed depending on both irradiation source and cellular age-status.

Across all conditions, PBM induced a dose-dependent increase in metabolic activity, with a maximal response observed at 5 J/cm^2^. In young cells, mean OD values increased from 0.495 ± 0.015 SEM in control cultures to 0.887 ± 0.006 SEM following LED irradiation at 5 J/cm^2^ and 0.771 ± 0.009 SEM following NIR irradiation at the same fluence. Similarly, in old cells, OD values increased from 0.673 ± 0.005 SEM in controls to 1.218 ± 0.017 SEM after LED irradiation and 0.903 ± 0.005 SEM following NIR irradiation at 5 J/cm^2^.

Post hoc comparisons using the Sidak multiple-comparison test confirmed that metabolic activity at 5 J/cm^2^ was significantly higher than at lower and higher fluences in all experimental groups (*p* < 0.0001), consistent with a biphasic PBM response. LED irradiation produced significantly higher OD values than NIR at the optimal doses (5 and 10 J/cm^2^) in both young and old cells, whereas NIR responses were modestly higher at the lowest fluence (0 J/cm^2^).

Comparison between age groups further revealed that old cells exhibited consistently higher metabolic activity than young cells following PBM, particularly under LED irradiation, where the difference between age groups was significant across all tested doses (*p* < 0.0001). Under NIR irradiation, age-related differences were also observed but were less pronounced, with no significant difference detected at 2.5 J/cm^2^.

Overall, these findings demonstrate that PBM enhances metabolic activity in C2C12 cells in a fluence-dependent manner, with maximal stimulation at 5 J/cm^2^ and a stronger response to LED compared with NIR irradiation. The magnitude of the response was consistently greater in replicatively aged cells, indicating increased sensitivity of older myoblasts to PBM stimulation. (Supplementary Table S3).

#### 2.2.2. Photobiomodulation Modestly but Significantly Increases ATP Production in C2C12 Cells

Intracellular ATP levels were quantified using a luciferase-based luminescence assay following irradiation with 660 nm LED or 830 nm NIR at 5 J/cm^2^ in young (≤5 passages) and old (≥30 passages) C2C12 cells (Figure 2B). Because ATP measurements were obtained using a luminescence assay (ATP-lite), which operates within a relatively narrow dynamic range, the resulting differences appear visually small but represent reproducible and statistically significant changes in cellular bioenergetics.

A factorial two-factor ANOVA (irradiation condition: control, LED, NIR × cell age: young vs old) revealed significant main effects of irradiation source (F_2,30_ ≈ 72.6, *p* < 0.0001) and cellular age (F_1,30_ ≈ 39.0, *p* < 0.0001), as well as a significant Source × Age interaction (F_2,30_ ≈ 8.9, *p* < 0.001), indicating that the ATP response to photobiomodulation differed modestly between young and old cells.

Post hoc comparisons (Sidak multiple-comparison test) showed that both LED and NIR irradiation significantly increased ATP levels relative to the control in young cells. Mean ATP levels increased from 3.626 ± 0.003 SEM in control cells to 3.645 ± 0.004 SEM following LED irradiation and 3.652 ± 0.003 SEM following NIR irradiation. These correspond to increases of approximately 0.52% (LED) and 0.71% (NIR) relative to the control.

In old cells, ATP levels increased from 3.593 ± 0.003 SEM in controls to 3.637 ± 0.002 SEM after LED irradiation and 3.642 ± 0.005 SEM after NIR irradiation, corresponding to increases of approximately 1.21% (LED) and 1.36% (NIR) relative to the control.

These results confirm that photobiomodulation induces a modest but statistically significant increase in cellular ATP production, with a slightly greater magnitude of response observed in aged cells.

Notably, the apparent similarity of the bars in young cells reflects the limited dynamic range of luminescence measurements rather than the absence of biological differences. Quantitative analysis of the underlying values confirmed statistically significant increases following PBM despite the small absolute magnitude of change.

#### 2.2.3. PBM Strongly Increases ROS Production Independent of Cellular Age

Reactive oxygen species (ROS) levels were assessed using a fluorescence-based ROS assay following irradiation with LED or NIR at 5 J/cm^2^ (Figure 2C).

Factorial two-way ANOVA (Source × Age) demonstrated a highly significant main effect of irradiation source (F_2,12_ = 295.15, *p* < 0.0001), indicating strong PBM-induced ROS generation. A smaller but statistically significant effect of cellular age was also observed (F_1,12_ = 4.84, *p* = 0.048). The Source × Age interaction was not significant (F_2,12_ = 1.73, *p* = 0.219), indicating that the magnitude of PBM-induced ROS generation was comparable between young and old cells.

In young cells, ROS-positive cells increased dramatically from 1.41 ± 0.81 SEM in control cultures to 82.77 ± 9.29 SEM following LED irradiation and 84.98 ± 3.46 SEM following NIR irradiation. Similarly, in old cells, ROS levels increased from 0.013 ± 0.002 SEM in controls to 93.82 ± 2.94 SEM following LED irradiation and 98.19 ± 0.66 SEM following NIR irradiation.

Post hoc comparisons confirmed that both LED and NIR irradiation produced highly significant increases in ROS production relative to the control in both young and old cells (*p* < 0.0001), while ROS levels did not differ significantly between LED and NIR treatments within the same age group.

These results indicate that PBM induces a robust oxidative signaling response that is largely independent of replicative aging in C2C12 cells.

### 2.3. Extracellular Vesicle Release and Characterization Following PBM

Nanoparticle tracking analysis (NTA) was used to quantify extracellular vesicle (EV) release from early-passage (≤5) and late-passage (≥30) C2C12 myoblasts following photobiomodulation at 5 J/cm^2^ using either 660 nm LED or 830 nm NIR irradiation. Extracellular vesicles comprise heterogeneous membrane-bound particles released by cells and are commonly classified according to their size and biogenesis into exosomes (~30–150 nm), microvesicles (~100–1000 nm), and apoptotic bodies (>500 nm). Small EV populations are typically characterized by the presence of tetraspanin membrane proteins such as CD9, CD63, and CD81, which are widely used as canonical markers of EV identity and origin [19,35]. Representative size distributions revealed vesicle populations predominantly within the 50–200 nm range across all experimental conditions (Figure S1), consistent with the enrichment of small EV populations.

**Figure 3:**
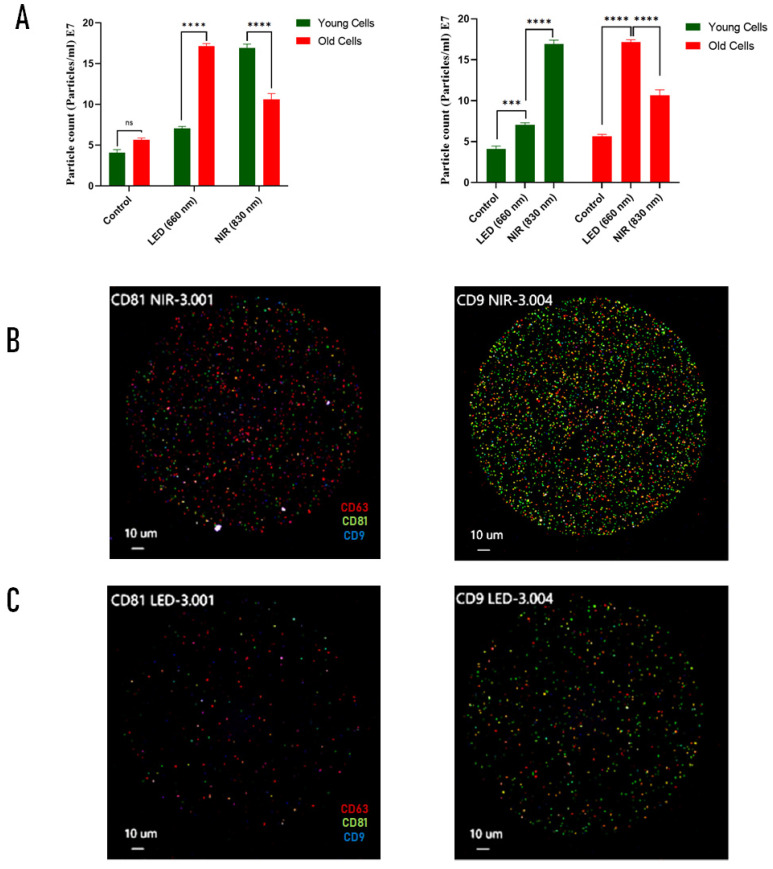
Source- and age-dependent regulation of extracellular vesicle (EV) release following photobiomodulation (PBM) in C2C12 cells. EVs were isolated from young (≤5 passages) and old (≥30 passages) C2C12 cells after irradiation with 660 nm LED or 830 nm NIR at 5 J/cm^2^. (**A**) EV particle concentration measured by nanoparticle tracking analysis (NTA) showing wavelength- and age-dependent differences in EV release. (**B**) Representative ExoView fluorescence micrographs of EVs captured from NIR-treated young cells using antibodies against the canonical tetraspanin markers CD81 and CD9 with co-detection of CD63. (**C**) Representative ExoView fluorescence micrographs of EVs captured from LED-treated young cells under the same conditions. Data in panel (**A**) represent mean ± SEM from three independent experiments. Statistical comparisons were performed using two-way ANOVA (treatment × age) followed by Tukey’s post hoc test. ns, not significant; *** *p* < 0.001; **** *p* < 0.0001. Color coding: in (**A**), green, young cells; red, old cells. In (**B**,**C**), red, CD63; green, CD81; blue, CD9.

Both light sources significantly increased EV particle counts relative to non-irradiated controls in young and old cells (Figure 3A, *p* < 0.001). In early-passage cells, NIR irradiation induced the highest EV yield, whereas in late-passage cells, LED treatment resulted in a comparatively greater increase in EV particle counts than NIR exposure, indicating an age-dependent shift in photonic source sensitivity.

To further assess qualitative differences in vesicle subpopulations, surface tetraspanin profiling was performed on EVs derived from young cells, which display higher metabolic and secretory activity and therefore provide a sensitive system for detecting source-specific differences in EV biogenesis. ExoView analysis confirmed the presence of canonical EV markers CD9, CD63, and CD81, while negative controls showed no detectable signal (Figure 3B,C and Figure S2).

Quantitative ExoView analysis revealed increased CD9- and CD81-positive particle counts following both LED and NIR treatments with NIR irradiation producing a higher proportion of triple-positive CD63/CD81/CD9 vesicles (Table 1 and Figure S2). These findings indicate that, in metabolically active myoblasts, NIR-based PBM preferentially enhances the release of canonical small EV populations.

### 2.4. Effects of PBM on Migration and Differentiation Across Replicative C2C12 Cells

**Figure 4:**
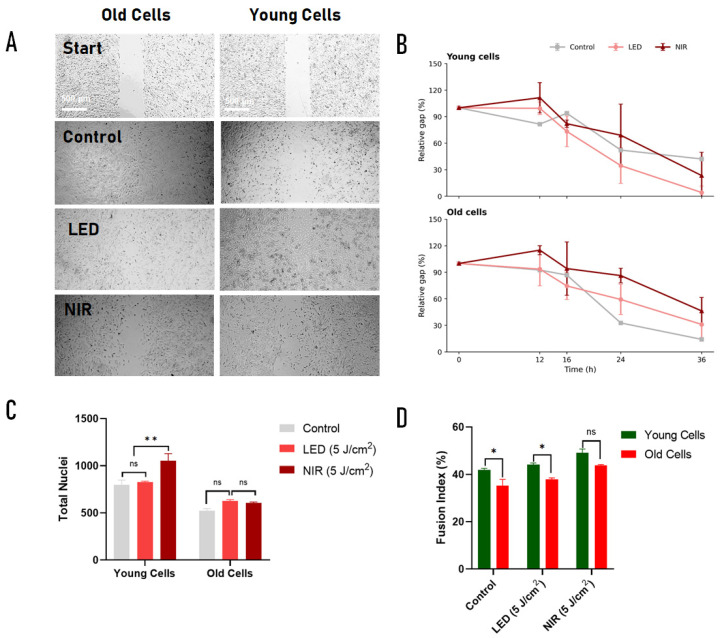
Photobiomodulation effects on migration and myogenic differentiation in young and replicatively aged C2C12 cells. (**A**) Representative bright-field images of the wound-healing assay in young (≤5 passages) and old (≥30 passages) C2C12 cells under control conditions or after irradiation with 660 nm LED or 830 nm NIR (5 J/cm^2^). Images were acquired 36 h after scratch induction. Scale bar: 500 µm. (**B**) Relative wound gap (%) over time (0–36 h), normalized to the initial gap (100%). (**C**) Total nuclei quantified after myogenic differentiation under control, LED, or NIR conditions. (**D**) Fusion index (%) of differentiated cultures in young and old cells. Data are mean ± SEM from three independent experiments (n = 3). Migration kinetics were analyzed using mixed-design ANOVA with time as the within-subject factor and age and treatment as between-subject factors, followed by Sidak multiple comparisons. Differentiation endpoints were analyzed using two-way ANOVA with Tukey post hoc test. ns, not significant; * *p* < 0.05; ** *p* < 0.01.

Cell migration was evaluated using a wound-healing assay in young (≤5 passages) and replicatively aged (≥30 passages) C2C12 cells following PBM with 660 nm LED or 830 nm NIR at 5 J/cm^2^, and wound closure was quantified over time (0–36 h) (Figure 4A).

Migration kinetics were analyzed using a mixed-design ANOVA with time as the within-subject factor and cell age (young vs old) and treatment (control, LED, NIR) as between-subject factors. The analysis revealed a significant main effect of time (F(4,48) = 149.783, *p* < 0.0001), indicating progressive wound closure across all groups. A significant main effect of treatment was also detected (F(2,12) = 4.963, *p* = 0.0269), whereas the main effect of age was not significant (F(1,12) = 0.822, *p* = 0.3825).

Importantly, a significant treatment × time interaction was observed (F(8,48) = 4.287, *p* = 0.0006), indicating that migration dynamics differed between PBM conditions over time. In addition, the age × treatment × time interaction was significant (F(8,48) = 3.323, *p* = 0.0042), demonstrating that the temporal response to PBM varied according to the replicative age of the cells. In contrast, the age × time interaction (F(4,48) = 0.411, *p* = 0.7997) and the age × treatment interaction (F(2,12) = 2.105, *p* = 0.1646) were not significant.

Descriptive analysis showed that in young cells, LED-treated cultures exhibited faster gap closure compared with control and NIR groups at later time points, with mean relative gap values decreasing from approximately 99.4 ± 7.0% at 12 h to 4.2 ± 7.3% at 36 h. Control cultures reached 42.1 ± 0.6% at 36 h, whereas NIR-treated cells showed intermediate closure kinetics (23.4 ± 26.1%).

In old cells (Figure 4B), migration dynamics differed from those observed in young cultures. Control cells showed progressive closure from 92.5 ± 1.2% at 12 h to 14.2 ± 0.5% at 36 h. LED-treated cells showed moderate closure (31.1 ± 15.7% at 36 h), while NIR-treated cultures maintained larger gap areas across time points, reaching 46.3 ± 15.3% at 36 h.

Post hoc pairwise comparisons using Sidak correction identified several significant differences between treatment conditions at specific time points. In young cells, significant differences were detected between the control and LED at 36 h (*p* = 0.0026) and between the control and NIR at 16 h (*p* = 0.024). In old cells, significant differences were observed between the control and NIR at 12 h (*p* = 0.0052) and at 24 h (*p* = 0.0011), as well as between the control and LED at 24 h (*p* = 0.043) and 36 h (*p* = 0.037).

Together, these results indicate that migration progressed over time in all groups but that the magnitude and trajectory of wound closure differed depending on PBM modality and cellular replicative age.

Interval-specific closure analysis revealed that the most rapid migration occurred between 12 and 16 h in both young and old cells, followed by a gradual decline in closure rate during subsequent intervals. In young cells, NIR showed slightly higher closure rates during the early post-widening phase (12–16 h), whereas LED promoted greater closure during the mid-phase of migration (16–24 h). In old cells, both PBM modalities showed transient increases in early interval closure rates, but these effects were not sustained and did not translate into improved overall wound closure compared with controls at later stages.

Differentiation capacity was assessed by calculating the fusion index, defined as the proportion of nuclei incorporated into multinucleated myotubes. Representative images combining bright-field visualization with Hoechst-stained nuclei (Figure S3) revealed increased multinucleation in PBM-treated groups compared with controls, with the most prominent effect observed under NIR irradiation in both young and old cells.

Quantification of total nuclei per field in the case of NIR treatment significantly increased total nuclei in young cells but not in old cells (Figure 4D, upper panel), consistent with a modest age-dependent response under differentiation-inducing conditions. Overall, nuclei counts remained within a similar range across treatments, reflecting near-confluent cultures maintained in low-serum medium to favor differentiation over proliferation.

In contrast, fusion index analysis demonstrated marked age- and treatment-dependent effects (Figure 4D). In old cells, the fusion index increased from 35.2 ± 4.7% in controls to 38.0 ± 1.1% with LED and 43.9 ± 0.5% with NIR. Statistical comparisons showed a significant improvement with NIR versus the control (*p* = 0.033) and a highly significant increase with NIR versus LED (*p* < 0.001), whereas LED alone did not significantly differ from the control (*p* = 0.377).

In young cells, the fusion index increased from 42.0 ± 1.0% in controls to 44.1 ± 1.3% with LED and 49.1 ± 2.8% with NIR. NIR significantly enhanced fusion relative to both the control (*p* = 0.014) and LED (*p* = 0.047), while LED treatment alone showed only a nonsignificant trend toward improvement compared with the control (*p* = 0.094).

Together, these data indicate that PBM, particularly NIR irradiation, robustly enhances myogenic fusion in both young and aged C2C12 myoblasts, whereas effects on total cell number are minimal or age-dependent under differentiation-inducing conditions. (Supplementaty Table S3).

## 3. Discussion

### 3.1. Responses to PBM

This study examined how photobiomodulation (PBM) delivered by two commonly used light sources, 660 nm LED and 830 nm NIR laser, modulates mitochondrial, metabolic, and functional responses in C2C12 myoblasts across replicative aging stages. Across multiple experimental scales, PBM consistently activated mitochondrial-linked signaling pathways, with 5 J/cm^2^ emerging as the most effective fluence for stimulating mitochondrial membrane potential (ΔΨm), metabolic activity, ATP production, extracellular vesicle (EV) release, and myogenic differentiation. Factorial statistical analysis further confirmed that PBM responses were jointly shaped by wavelength, dose, and replicative age, indicating that the observed biological outcomes arise from the interaction of these variables rather than from a single experimental factor.

At the single-cell level, PBM induced a dose-dependent increase in mitochondrial membrane potential and ROS signaling, with maximal responses observed at 5 J/cm^2^ followed by saturation at higher fluence. This pattern reflects acute mitochondrial activation consistent with photon absorption by cytochrome c oxidase, which enhances electron transport and transiently elevates ΔΨm and signaling ROS [3,36,37,38]. These short-lived ROS pulses are consistent with mitohormetic signaling, where moderate oxidative bursts function as second messengers that activate adaptive transcriptional and metabolic programs rather than inducing damage [7,8,38]. Importantly, the single-cell assays showed a dose-dependent increase followed by a plateau, whereas the population-level metabolic assays displayed a more classical biphasic response, with maximal stimulation at 5 J/cm^2^ followed by reduced responses at higher doses. This difference likely reflects the transition from immediate mitochondrial signaling in individual cells to integrated metabolic outcomes at the population level [15,39]. The single-cell screening experiments were performed in intermediate-passage (P25) cells to provide a stable system for fluence optimization prior to the age-stratified experiments conducted in young (≤5 passages) and aged (≥30 passages) cultures.

Replicative aging significantly influenced PBM responsiveness. Young (≤5 passages) and aged (≥30 passages) C2C12 cells both exhibited increased ROS signaling following irradiation, while ATP production increased modestly but significantly after PBM. In aged cells, both LED and NIR increased ATP levels, although LED produced a slightly larger increase, indicating an age-dependent difference in energetic sensitivity rather than a reduction under NIR. These modest but reproducible increases are consistent with mitochondrial activation inferred from ΔΨm, ATP, and ROS responses rather than direct measurements of respiratory activity [3,36,37,38]. Replicative aging models of C2C12 cells are known to display impaired mitochondrial efficiency, altered redox balance, and reduced differentiation capacity [33,34,40]. In the present work, senescence-associated β-galactosidase staining confirmed increased cellular aging in late-passage cultures, supporting the biological distinction between the young and aged conditions used in the experiments.

Functional assays revealed that PBM influenced migration kinetics but did not produce sustained enhancement of wound closure, particularly in replicatively aged cells. In contrast, myogenic differentiation was strongly enhanced by NIR irradiation, as reflected by increased fusion index in both young and aged cultures. These findings suggest that PBM primarily supports late-stage myogenic programs, likely through improved mitochondrial polarization, ATP availability, and ROS-mediated signaling pathways involved in myogenic transcription and cytoskeletal remodeling [18,41,42]. Transient ROS signaling is known to activate pathways such as NF-κB and MAPK that promote differentiation when maintained within physiological limits [24,34,43,44]. The stronger differentiation response observed with NIR irradiation may therefore reflect more efficient coupling between mitochondrial activation and downstream myogenic signaling programs.

PBM also significantly enhanced extracellular vesicle release, with source- and age-dependent differences in EV secretion. In young myoblasts, NIR irradiation produced the highest EV yields and increased the proportion of canonical small EV populations characterized by CD9, CD63, and CD81 expression. EV biogenesis is closely linked to mitochondrial activity, intracellular calcium signaling, and redox regulation, all of which are influenced by PBM [39,45]. Tetraspanin profiling was performed only in young cells because their higher metabolic activity and more homogeneous EV populations provide a sensitive system for resolving subtle changes in vesicle phenotype. In aged cells, EV secretion increased more strongly after LED irradiation, suggesting that replicative aging alters membrane trafficking and vesicle biogenesis pathways. These findings indicate that PBM may influence not only direct cellular metabolism but also paracrine signaling through EV release, potentially contributing to regenerative communication networks [46].

Differences between LED and NIR irradiation likely arise from a combination of optical and biological factors. Although both light sources were calibrated to deliver equivalent fluence at the culture plane, lasers and LEDs differ in coherence, spectral bandwidth, and beam distribution. Under the controlled dosimetry conditions used here, both sources produced comparable mitochondrial activation but diverged at higher-order functional outputs. LED irradiation preferentially enhanced metabolic activity and ATP production, whereas NIR more consistently promoted myogenic differentiation. EV responses were age-dependent, with NIR favoring EV release in young cells and LED eliciting the greater increase in aged cells. These findings support the concept that PBM outcomes are shaped not only by wavelength but also by source-specific optical delivery characteristics, which can influence how mitochondrial signals are translated into functional cellular responses [25,30,47,48].

### 3.2. Limitations

Several limitations should be considered when interpreting these findings. First, the experiments were conducted in an in vitro C2C12 myoblast model, which cannot fully reproduce the structural, vascular, and immune complexity of skeletal muscle tissue. Second, mitochondrial activation was inferred from ΔΨm, ATP, and ROS measurements rather than direct assessment of respiratory parameters such as oxygen consumption rate or cytochrome c oxidase activity. Third, the single-cell mitochondrial screening experiments were performed in intermediate-passage (P25) cells to establish a stable platform for fluence optimization prior to the age-stratified analyses. Although subsequent population-level experiments confirmed that 5 J/cm^2^ produced consistent responses in both young and aged cells, single-cell responses were not evaluated across the full replicative aging spectrum. Fourth, differences in optical configurations between the single-cell micro-irradiation system and bulk culture irradiation platforms resulted in different irradiance conditions despite matched fluence, which may influence how PBM signals are distributed within cells. Finally, extracellular vesicle phenotyping was performed only in young cells, and future studies should examine EV subpopulations in aged cells to better understand age-dependent changes in PBM-regulated vesicle biogenesis. Additional in vivo and mechanistic studies will therefore be required to determine whether the mitochondrial, redox, and differentiation responses observed here translate into sustained improvements in muscle regeneration and function.

## 4. Materials and Methods

**Figure 5:**
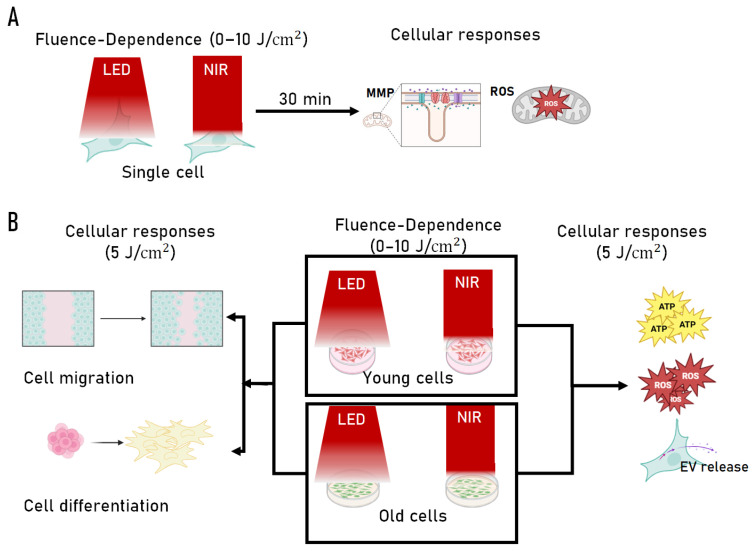
Roadmap of the photobiomodulation (PBM) experimental design. (**A**) Single-cell experimental workflow illustrating fluence-dependent PBM exposure (0–10 J/cm^2^) using either a 660 nm LED or an 830 nm NIR laser, followed by quantitative assessment of mitochondrial membrane potential (ΔΨm, reported as MMP) and reactive oxygen species (ROS) at the individual cell level (passage 25). (**B**) Population-level experimental design in which C2C12 myoblasts representing different replicative ages, defined as young (≤5 passages) and old (≥30 passages), were exposed to source-specific PBM across the same fluence range, with functional outcomes assessed at an optimized fluence (5 J/cm^2^), including ATP production, ROS levels, extracellular vesicle (EV) release, cell migration, and myogenic differentiation. Together, this roadmap summarizes the integrated strategy linking light source, fluence, and replicative cellular age to mitochondrial and regenerative responses. Arrows indicate the experimental workflow and direction of analysis.

Figure 5 provides a schematic overview of the experimental design, illustrating the single-cell dose-screening strategy and the subsequent population-level and functional assays performed in young and replicatively aged C2C12 myoblasts. Detailed methods for each experimental step are described below.

### 4.1. Cell Culture

C2C12 murine myoblasts (BCRC No. 60083; Bioresource Collection and Research Center, Hsinchu, Taiwan), originally derived from the C3H mouse lineage, were used in this study. The working cell stocks were kindly provided by Professor Yi-Fan Chen (College of Medical Science and Technology, Taipei Medical University) and Dr. Aline Yen-Ling Wang (Center for Vascularized Composite Allotransplantation, Chang Gung Memorial Hospital). According to the supplier’s certification, the cells were confirmed to be free of mycoplasma contamination. Cells were cultured in Dulbecco’s Modified Eagle Medium (DMEM; Gibco, Thermo Fisher Scientific, Waltham, MA, USA; Cat#11965-084) without sodium pyruvate, supplemented with 10% fetal bovine serum (FBS; Gibco, Thermo Fisher Scientific, Waltham, MA, USA) and 1% penicillin–streptomycin. Cultures were maintained at 37 °C in a humidified 5% CO_2_ atmosphere. Cells were routinely passaged at 50–60% confluence to maintain a proliferative myoblastic phenotype and avoid spontaneous differentiation [34,49]. Young cells were defined as passage ≤ 5, while old cells were defined as passage ≥ 30, consistent with established models of replicative aging in myoblasts [18,33,34,41,50,51].

Cryopreservation was performed in complete medium with 5% dimethyl sulfoxide (DMSO). Cells were stored in liquid nitrogen and thawed by rapid warming at 37 °C, followed by centrifugation at 200× *g* for 5 min and resuspension in fresh medium. Differentiation was induced at 90% confluence by switching to DMEM supplemented with 2% horse serum (non-heat inactivated; Gibco Ref.16050-122) and refreshing medium daily [52,53].

#### Senescence-Associated β-Galactosidase Staining

Senescence-associated β-galactosidase (SA-β-gal) staining was performed to qualitatively evaluate replicative senescence in early- and late-passage C2C12 cultures. Cells were stained using a commercial Cell Senescence β-Galactosidase Staining Kit (Cat. No. HY-K1089; MedChemExpress, Monmouth Junction, NJ, USA) according to the manufacturer’s instructions. This assay detects senescence-associated β-galactosidase activity using the chromogenic substrate X-gal, which is cleaved by β-galactosidase to produce an insoluble blue precipitate in senescent cells that can be visualized by bright-field microscopy.

Briefly, C2C12 cells at different passages were washed with PBS, fixed with the provided fixation solution, and incubated with the β-galactosidase staining working solution at 37 °C under CO_2_-free conditions until blue staining developed. Cells were then examined using bright-field microscopy, and representative images were acquired to qualitatively assess the presence of senescent cells. Increased blue staining was observed in late-passage cultures compared with early-passage cells. Representative images are shown in Supplementary Figure S4.

### 4.2. Photobiomodulation (PBM) Irradiation Systems

**Figure 6:**
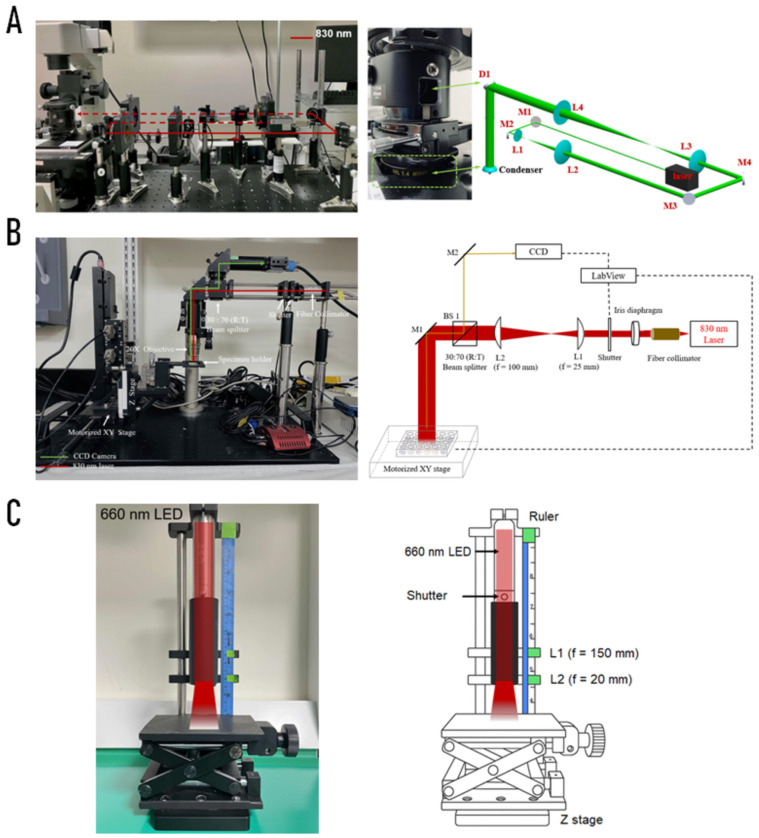
Experimental photobiomodulation (PBM) irradiation systems. (**A**) Custom-built 830 nm near-infrared (NIR) laser platform integrated with an inverted Nikon TE2000U microscope for single-cell irradiation and fluorescence imaging, with schematic of the optical path showing plano-convex lenses (L1–L4), mirrors (M1–M4), and dichroic mirror (D1). (**B**) Bulk-cell 830 nm NIR irradiation system used for viability and functional assays, consisting of a fiber-coupled laser diode, collimation optics (L1, L2), iris diaphragm, mechanical shutter, 30:70 (R:T) beam splitter, and CCD monitoring system mounted on a motorized XY stage, with schematic of beam delivery to multiwell plates. (**C**) Customized 660 nm LED irradiation system designed for uniform illumination, comprising a vertically mounted LED source, beam-shaping optics (L1 = 150 mm, L2 = 20 mm), mechanical shutter, and adjustable Z-stage, enabling stable and homogeneous fluence delivery across microchambers and multiwell culture formats. Dashed lines indicate optical paths and system connections, including signal acquisition and control interfaces.

Three complementary PBM platforms were employed in this study (Figure 6), consisting of two near-infrared (NIR) laser sources at 830 nm and one red light-emitting diode (LED) source at 660 nm. The 830 nm NIR laser (Lambda Beam Wavelock, RGB Photonics, Bavaria, Germany) integrated with the inverted microscope (TE2000U; Nikon Corporation, Tokyo, Japan) was configured for single-cell microchamber irradiation combined with live-cell imaging. A separate 830 nm NIR laser optical system (MLL-III-830 nm-2 W-EK10935; Oceanhood, Taoyuan, Taiwan), not coupled to the microscope, was used for controlled irradiation of cell cultures at the population level. In addition, the 660 nm LED platform (Holistic Technologies LLC, Milton, MA, USA) integrated with a custom optical assembly (Oceanhood, Taoyuan, Taiwan) was configured for both single-cell microchamber irradiation and bulk population exposure in multiwell plate formats (96-well and 24-well plates).

For all systems, delivered fluence was calculated from the measured output power, exposure duration, and illuminated area using the standard relationship [54]:

(1) $$Fluence\;J/{cm}^{2}=\frac{power\;(W)\;\times \;time\;(s)\;}{area\;({cm}^{2})}$$

#### 4.2.1. PBM Dose Selection and Experimental Design

For both the 830 nm NIR laser and the 660 nm LED platforms, fluence ranges were selected according to the specific experimental format and biological endpoint, following a stepwise dose-screening strategy.

Single-cell experiments (microchamber platforms): For single-cell irradiation combined with live fluorescence imaging, three fluences were tested for both light sources: 2.5, 5.0, and 10.0 J/cm^2^. These experiments were designed to assess immediate mitochondrial membrane potential and redox responses within the same cell before and after PBM exposure, enabling identification of source-specific and dose-dependent mitochondrial effects at the individual cell level.

Bulk cell experiments (multiwell formats). For population-level assays, fluence ranges were adapted to the specific readout (Supplementary Table S1):

- Cell viability screening (96-well format). To more fully characterize biphasic dose–response behavior, bulk viability assays were performed using 2.5, 5.0, 10.0, and 15.0 J/cm^2^ for both LED and NIR platforms.
- Mechanistic and functional assays (96- and 24-well formats). Based on concordant findings from single-cell mitochondrial assays and bulk viability screening, 5.0 J/cm^2^ was identified as the most consistently effective fluence across both light sources. This dose was therefore selected for all subsequent functional studies, including ATP production (96-well), ROS signaling, migration, differentiation, and extracellular vesicle (EV) release (24-well format).

##### Dosimetry and Irradiation Control

For each optical configuration, beam geometry and alignment were fixed. Equivalent fluences were achieved by adjusting exposure time and illuminated area, while maintaining constant source output within each configuration. Fluence modulation was therefore accomplished exclusively through changes in exposure duration, as detailed in Table 2. Microchamber conditions correspond to confined irradiation for single-cell analyses, whereas 96-well and 24-well formats represent bulk population exposure.

Real-time light output for each irradiation system was verified using a wavelength-adjusted thermal power sensor (PD300-3W-V1, Ophir, Israel), with calibration performed at the sample plane after accounting for optical transmission factors and confirmed before each experiment to ensure irradiance stability and reproducibility across experimental sessions.

All irradiations were conducted at ambient laboratory temperature (25 °C), with culture plates removed from the incubator only for the duration of light exposure to minimize environmental fluctuations. Irradiations were performed under controlled ambient lighting conditions, with overhead laboratory lights switched off so that samples were exposed exclusively to the designated PBM source, thereby avoiding unintended background photostimulation.

During LED-based experiments, only the target well or microchamber being irradiated was exposed at any given time, while all surrounding wells and non-treated samples, including control groups, were shielded with opaque covers to prevent exposure to scattered or reflected light during neighboring irradiations. The plate layout was arranged to avoid immediate adjacency of experimental and control conditions.

#### 4.2.2. Single-Cell NIR Laser System (830 nm)

For single-cell experiments assessing mitochondrial membrane potential (ΔΨm/MMP) and reactive oxygen species (ROS), a custom-built 830 nm continuous-wave NIR laser platform was integrated into a Nikon TE2000U inverted microscope (Nikon Corporation, Tokyo, Japan) [36]. The collimated laser beam was expanded through a series of plano-convex lenses (L1–L4) and directed into the optical path by a dichroic mirror (D1). Mirrors (M1–M4) ensured alignment and beam delivery into a microfluidic chamber containing adherent C2C12 cells. To achieve photobiomodulation-relevant irradiance levels for single-cell experiments, the laser output was attenuated using a reflective neutral density filter (ND4; Thorlabs), reducing the transmitted power to approximately 0.01% of the original output before reaching the micro-irradiation area. This configuration enabled localized, high-resolution irradiation and real-time single-cell fluorescence imaging (Figure 6A).

The illuminated area used for single-cell NIR irradiation was experimentally determined from the beam profile at the sample plane and measured in ImageJ (version 1.54g; National Institutes of Health, Bethesda, MD, USA). [36]. The resulting micro-irradiation area was approximately 800 µm^2^ (8.0 × 10^−6^ cm^2^), which was selected to approximate the projected area of a single adherent C2C12 myoblast.

In contrast to the LED configuration, the NIR laser system was focused to irradiate individual cells sequentially, enabling direct single-cell light delivery and post-irradiation imaging of the same targeted cells.

#### 4.2.3. Bulk-Cell NIR Laser System (830 nm)

For population-level experiments (cell viability, migration, differentiation, and extracellular vesicle release), a separate 830 nm NIR laser system was employed [39]. The setup consisted of a fiber-coupled laser diode, collimation optics (L1, L2), iris diaphragm, and mechanical shutter. A 30:70 beam splitter directed part of the beam toward a CCD monitoring system, while the main beam illuminated cells cultured in 96 or 24 well plates mounted on a motorized XY stage. This arrangement allowed uniform dosing across multiple wells and facilitated automated treatment protocols (Figure 6B).

#### 4.2.4. Customized LED System (660 nm)

For comparison with laser irradiation, a custom collimated LED system (660 nm) was constructed for photobiomodulation experiments, with optical conditioning inspired by the design reported by Baldassarro et al. [37] to ensure controlled irradiance, uniform illumination, and reproducibility. The LED source was mounted in a fixed metallic holder at the top of a vertical optical rail and remained stationary throughout all experiments (Oceanhood Taoyuan, Taiwan). Beam shaping and divergence control were achieved using two movable plano-convex lenses (L1 = 150 mm, L2 = 20 mm) mounted on adjustable carriers along the same rail [54].

An optical pinhole with a 5 mm diameter (ID12; Thorlabs, Newton, NJ, USA) was inserted when required to further constrain the beam profile for microchamber and 96-well plate irradiation. In the case of microchamber experiments, the pinhole was positioned immediately before L1, whereas for 96-well plate irradiation, it was positioned immediately before L2. Biological samples were placed on an adjustable Z-stage located beneath the optical train, allowing precise positioning of microchambers, 96-well plates, or 24-well plates at the desired focal distance. By adjusting the relative positions of L1, L2, and the Z-stage while keeping the LED source fixed, the illuminated field could be matched to the required treatment area of each experimental format, ensuring consistent fluence delivery across both microchambers and culture plates (Table 2).

Exposure timing was controlled using a mechanical shutter, and fixed optical alignment ensured stable and repeatable irradiation geometry across experiments. This system was therefore used for both single-cell mitochondrial measurements and population-level functional assays, including viability, migration, differentiation, and extracellular vesicle analysis (Figure 6C).

For single-cell analyses under LED irradiation, the entire central microchamber field (0.196 cm^2^) was uniformly illuminated, with mitochondrial and redox parameters subsequently quantified at the individual cell level (same cell pre- and post-treatment) by inverted fluorescence microscopy within the treated area.

Irradiance stability and spatial uniformity of the 660 nm LED output were monitored using an Ophir Starlite power meter to ensure consistent exposure conditions across experiments.

### 4.3. Single-Cell Assays of Mitochondrial Function and ROS

Single-cell mitochondrial screening experiments were performed in intermediate-passage C2C12 cells (P25), which served as a stable platform for fluence optimization prior to the age-stratified population-level experiments performed in early- and late-passage cultures.

#### 4.3.1. Mitochondrial Membrane Potential (ΔΨm)

Mitochondrial membrane potential (ΔΨm, MMP) was assessed using Rhodamine 123, a cationic fluorescent probe widely used to monitor changes in mitochondrial polarization in live cells [55,56,57]. Cells were incubated with Rhodamine 123 (Rh123; 5 µM, PromoCell GmbH) as previously described by Pan et al. [36] and validated as a ΔΨm probe [55,58]. Fluorescence images were acquired immediately before irradiation (S_1_) and 30 min after PBM exposure (S_2_). For each fluence condition, 15 individual cells were analyzed, resulting in a total of 60 cells across the four dose groups. For each analyzed cell, five fluorescence images were processed, yielding 300 images for quantitative analysis. Image processing was performed using a custom MATLAB-based workflow (MATLAB R2023b Update 7; MathWorks, Natick, MA, USA). Briefly, fluorescence images were imported into MATLAB, background noise was removed using radius-based background subtraction, and mitochondrial morphology was visually verified to ensure accurate signal extraction. Mean fluorescence intensity was then calculated for the irradiated cell region, and the mitochondrial membrane potential response was expressed as the post-/pre-irradiation fluorescence ratio (S_2_/S_1_).

#### 4.3.2. Intracellular ROS

Intracellular ROS were detected using H_2_DCFDA (10 µM; Invitrogen, Thermo Fisher Scientific, Waltham, MA, USA; Cat# D-39910) before irradiation (R_1_) and 30 min after PBM exposure (R_2_)**.** Fluorescence responses were expressed as R_2_/R_1_ ratios, following previously described analysis methods [36], with interpretation guided by established ROS-detection frameworks [56,59,60]. Imaging was performed using an EMCCD camera (LucaEM S658M; Andor Technology Ltd., Belfast, UK), and fluorescence intensity was quantified in MATLAB using local background subtraction, as previously described [36].

### 4.4. Population Level Experiments

#### 4.4.1. Cell Viability

Cell viability was determined using the Cell Counting Kit-8 (CCK-8; Elabscience, Wuhan, China; Cat# E-CK-A361) following published protocols [39]. C2C12 cells were seeded at 5 × 10^3^/well in 96-well plates and irradiated at 0, 2.5, 5, or 10 J/cm^2^ (LED or NIR). After 24 h, absorbance was measured at 450 nm using a microplate spectrophotometer (Epoch 2NS; Agilent BioTek Instruments, Inc., Winooski, VT, USA).

#### 4.4.2. Bulk ROS Measurement

Intracellular ROS levels in young (≤5 passages) and old (≥30 passages) C2C12 cells were assessed using the ROS-ID^®^ Total ROS Detection Kit (Enzo Life Sciences, Inc., Farmingdale, NY, USA; Cat# ENZ-51011). Cells were seeded at 1 × 10^5^ cells per well in 24-well plates and allowed to adhere overnight. Following PBM irradiation with LED (660 nm) or NIR (830 nm) at the selected fluence of 5 J/cm^2^, cells were incubated with 200 μL of ROS detection solution for 30 min, followed by the addition of 300 μL of ROS buffer (total 500 μL). Negative control wells received only ROS buffer without the detection dye, and positive controls received ROS inducer. Fluorescence was analyzed by flow cytometry (FACSDiscover™ S8; BD Biosciences, San Jose, CA, USA; Ex = 488 nm, Em = 520 nm) to quantify ROS-positive cells across treatment groups [54]. The gating strategy and representative plots for ROS detection are provided in Supplementary Figures S5 and S6.

#### 4.4.3. Bulk ATP Measurement

Cellular ATP content was measured using the ATPlite 1step Luminescence Assay System (Revvity, Inc., Waltham, MA, USA; Cat# 6016736). Young (≤5 passages) and old (≥30 passages) C2C12 cells were seeded at 5 × 10^4^ cells per well in 96-well plates and cultured overnight. After PBM irradiation with LED (660 nm) or NIR (830 nm) at 5 J/cm^2^, 100 μL of ATP detection reagent was added directly to each well according to the manufacturer’s protocol. Plates were shaken for 20 s to ensure adequate mixing, and luminescence was measured using a microplate reader. Relative ATP levels were normalized to the corresponding non-irradiated control groups, and results were interpreted in accordance with previously established ATP-based mitochondrial activity measurements [54,61,62].

#### 4.4.4. Migration Assay

Cell migration was evaluated using standardized culture-insert chambers (ibidi GmbH, Gräfelfing, Germany), which generate a reproducible 500 μm cell-free gap [39]. Inserts were positioned in the outer wells of a 24-well plate to match the irradiation parameters optimized for this plate format. C2C12 myoblasts were seeded at a density of 3 × 10^4^ cells per chamber side and allowed to form a confluent monolayer following 12 h incubation at 37 °C in 5% CO_2_. Inserts were then carefully removed to create the defined wound area, and cultures were exposed to PBM (LED or NIR) at a fluence of 5 J/cm^2^. To minimize the contribution of proliferation and isolate migration, cultures were maintained in serum-free DMEM immediately after PBM. At 12 h post-irradiation, the medium was replaced with low-serum DMEM containing 2% FBS for the remainder of the assay [63]. Bright-field images (4× objective) of gap closure were acquired at 0, 12, 16, 24, 36, and 48 h post-treatment using an inverted microscope (DMi8; Leica Microsystems GmbH, Wetzlar, Germany). Migration dynamics were quantified as percentage gap closure over time using ImageJ (version 1.54g; National Institutes of Health, Bethesda, MD, USA) [64] (by an investigator blinded to experimental conditions). All experiments were performed in triplicate to ensure reproducibility.

#### 4.4.5. Differentiation Assay

Differentiation was induced as previously described in the Section 4.1. Cells were subsequently stained with Hoechst 33342 (Thermo Fisher Scientific, Waltham, MA, USA) for nuclear visualization. For each condition, images were acquired in both bright-field and fluorescence (Hoechst) channels using identical exposure times and magnification settings across all groups. Bright-field images were used to evaluate myotube morphology, while co-registered Hoechst fluorescence images were used to delineate nuclei (Leica DMi8 manual inverted microscope, Germany). Multiple non-overlapping fields were analyzed per well across at least three independent experiments.

In each field, the total number of nuclei (N_total) was first determined. Multinucleated myotubes were then identified based on morphological criteria, defined as elongated fibers containing two or more Hoechst-positive nuclei within a continuous cytoplasm and clearly separated from adjacent cells. Nuclei located within these myotubes were counted as N_MT. The fusion index (FI) was calculated as:

(2) $$\mathrm{FI}\;(\%)\;=\frac{N\text{\_}MT}{N\text{\_}total}\times 100$$

All counts were performed by a blinded rater, and discrepancies were reconciled by joint review, in a protocol adapted from [18,65,66].

#### 4.4.6. Extracellular Vesicle Isolation and Characterization

For extracellular vesicle (EV) isolation, C2C12 myoblasts at early passage (P ≤ 5) or late passage (P ≥ 30) were seeded in 24-well tissue-culture plates at a density of 5 × 10^4^ cells per well and allowed to adhere overnight at 37 °C with 5% CO_2_. Conditioned medium was collected 24 h after PBM exposure (5 J/cm^2^ using either the 660 nm LED or 830 nm NIR laser system) under serum-free conditions. EVs were isolated by differential centrifugation followed by ultracentrifugation [39,45], with all steps performed at 4 °C. Supernatants were sequentially cleared at 200× *g* for 10 min, 2000× *g* for 10 min, and 10,000× *g* for 30 min, retaining the supernatant at each step. The final supernatant was then ultracentrifuged at 100,000× *g* for 70 min, and the EV pellet was resuspended in 0.1 µm-filtered PBS for downstream analyses and storage at −80 °C.

Particle size distribution and concentration were quantified by nanoparticle tracking analysis (NTA; NanoSight NS300, Malvern Panalytical Ltd., Malvern, UK) using a 532 nm laser. Instrument settings and acquisition duration were kept constant across all groups, and measurements were conducted at concentrations yielding 20 to 100 particles per frame to ensure robust tracking. Each sample was measured in technical triplicate using 60 s video acquisitions, and values were averaged. For each experimental condition, conditioned medium from three wells of a 24-well plate was pooled for NTA.

Surface tetraspanin profiling was performed using the ExoView R200 platform with the Leprechaun Exosome Mouse Tetraspanin Kit to detect CD9, CD63, and CD81. Sample input concentrations were adjusted to 1 × 10 ^7^–1 × 10 ^9^ EVs/mL based on NTA results. Chips were processed and analyzed according to the manufacturer’s instructions, and particle counts per capture spot as well as fluorescence-confirmed subpopulations were quantified. This platform was selected because it preserves vesicle integrity, enables single-particle resolution, and has been applied in PBM studies to confirm consistency in EV size and marker expression between PBM-treated and control samples [35].

The overall PBM-EV experimental framework was aligned with recent mechanistic studies of biophysical stimulation [67,68,69] and PBM-based tissue and cell models [39,45,54,70].

### 4.5. Statistical Analysis

All experiments were performed with ≥3 biological replicates. Data are presented as mean ± SEM. Single-cell dose–response data were analyzed using one-way ANOVA followed by Tukey’s post hoc test. Population-level metabolic screening experiments were analyzed using three-way factorial ANOVA (dose × wavelength × age) followed by Sidak multiple-comparison tests. ATP and ROS datasets were analyzed using two-way ANOVA (source × age) with Tukey post hoc comparisons. Migration time-course data were analyzed using mixed-design ANOVA with time as the within-subject factor and treatment and age as between-subject factors. Statistical significance was defined as *p* < 0.05. Graphs were created and statistical analyses were performed using GraphPad Prism 8 (GraphPad Software, LLC, San Diego, CA, USA).

## 5. Conclusions

This study demonstrates that photobiomodulation delivered at 660 nm (LED) and 830 nm (NIR) activates mitochondrial and redox signaling in C2C12 myoblasts in a fluence-dependent manner while producing distinct functional outcomes shaped by irradiation source and replicative age. Although both light sources induced comparable mitochondrial polarization and mitohormetic ROS signaling at the single-cell level, their downstream effects diverged at the population level. LED irradiation preferentially enhanced metabolic activity and ATP production, particularly in aged cells, whereas NIR irradiation more consistently promoted myogenic fusion and differentiation efficiency across age groups. These findings indicate that replicative stage influences how early mitochondrial signals are translated into functional cellular responses. Collectively, the results support the concept that photobiomodulation parameters and light sources should be tailored to specific biological objectives and suggest that age-adapted PBM strategies may enhance approaches for muscle regeneration research.

## Figures and Tables

**Table 1:** Quantitative analysis of EV tetraspanin colocalization detected by ExoView.

EV Subtype	LED-3 (E7 Particles/mL)	NIR-3 (E7 Particles/mL)
CD63	0.489 ± 0.048	1.016 ± 0.037
CD81	1.276 ± 0.095	3.084 ± 0.308
CD9	0.250 ± 0.025	0.344 ± 0.010
CD63/CD81	0.916 ± 0.059	4.701 ± 0.590
CD63/CD9	0.014 ± 0.006	0.047 ± 0.003
CD81/CD9	0.131 ± 0.021	0.528 ± 0.039
CD63/CD81/CD9	0.398 ± 0.064	1.451 ± 0.091

**Table 2:** Photobiomodulation dosimetry parameters for single-cell and bulk irradiation formats using 660 nm LED and 830 nm NIR laser sources. Single-cell microchamber experiments were performed at 2.5, 5, and 10 J/cm^2^ to evaluate dose-dependent mitochondrial responses. Bulk viability assays in 96-well plates included an extended fluence range (2.5–15 J/cm^2^) to assess biphasic effects. Based on these dose-screening experiments, 5 J/cm^2^ was selected for subsequent functional assays in 24-well plates, including ROS, migration, differentiation, and extracellular vesicle analyses. Power output and illuminated area were constant within each optical configuration, and fluence was modulated by adjusting exposure time.

System and Light Source	Fluence (J/cm^2^)	Time (s)	Power (W)	Area (cm^2^)
Microchamber NIR 830 nm (Thorlabs ND40A)	2.5	27	7.3 × 10^−7^	8.0 × 10^−6^
	5.0	54		
	10.0	108		
Microchamber LED 660 nm (5 mm pinhole before L1)	2.5	27	0.0181	0.196
	5.0	54		
	10.0	108		
96-well NIR 830 nm	2.5	130	0.0073	0.38
	5.0	260		
	10.0	520		
	15.0	780		
96-well LED 660 nm (5 mm pinhole before L2)	2.5	28	0.034	0.38
	5.0	56		
	10.0	112		
	15.0	168		
24-well NIR 830 nm	5.0	32	0.311	2.01
24-well LED 660 nm	5.0	75	0.127	1.90

Note: Power values for the single-cell NIR configuration correspond to the effective power at the sample plane after attenuation with an ND4 neutral density filter (10^−4^ transmission).

## Data Availability

The original contributions presented in this study are included in the article/Supplementary Materials. Further inquiries can be directed to the corresponding authors.

## Supplementary Materials

The following supporting information can be downloaded at: https://www.mdpi.com/article/10.3390/ijms27072999/s1.

## Abbreviations

The following abbreviations are used in this manuscript:

ANOVA	Analysis of Variance
ATP	Adenosine Triphosphate
CCK-8	Cell Counting Kit-8
CcO	Cytochrome c Oxidase
CO2	Carbon Dioxide
DMEM	Dulbecco’s Modified Eagle Medium
DMSO	Dimethyl Sulfoxide
ΔΨm	Mitochondrial Membrane Potential
EMCCD	Electron-Multiplying Charge-Coupled Device
ESCRT	Endosomal Sorting Complex Required for Transport
EV	Extracellular Vesicle
FACS	Fluorescence-Activated Cell Sorting
FBS	Fetal Bovine Serum
FI	Fusion Index
LED	Light-Emitting Diode
MAPK	Mitogen-Activated Protein Kinase
MMP	Mitochondrial Membrane Potential
NIR	Near-Infrared
NTA	Nanoparticle Tracking Analysis
PBM	Photobiomodulation
Rh123	Rhodamine 123
ROS	Reactive Oxygen Species
SEM	Standard Error of the Mean
