# Supplementary material for: Source-Specific Photobiomodulation Regulates Mitochondrial Bioenergetics, Redox Signaling, and Functional Outputs in C2C12 Myoblasts Across Replicative Aging
Source: Int J Mol Sci. 2026 Mar 25;27(7):2999. doi: 10.3390/ijms27072999 (PMC13073692; doi:10.3390/ijms27072999)
Supplement: Supplementary file 1 [file ijms-27-02999-s001.zip › ijms-4155450-supplementary.pdf]

SUPPLEMENTARY MATERIAL

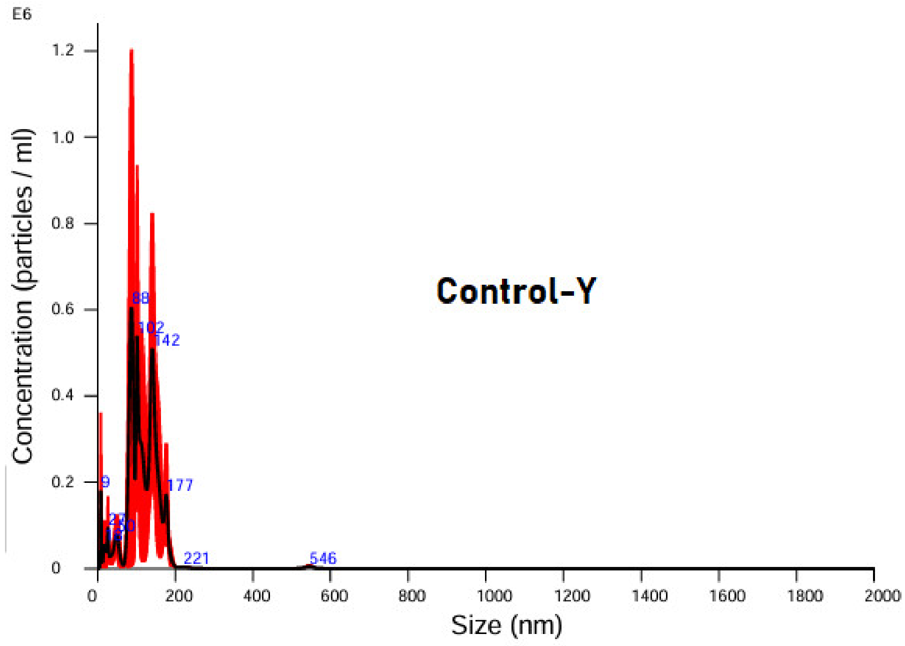
E6
Concentration (particles / ml)
Control-Y
88
102
142
177
221
546
Size (nm)

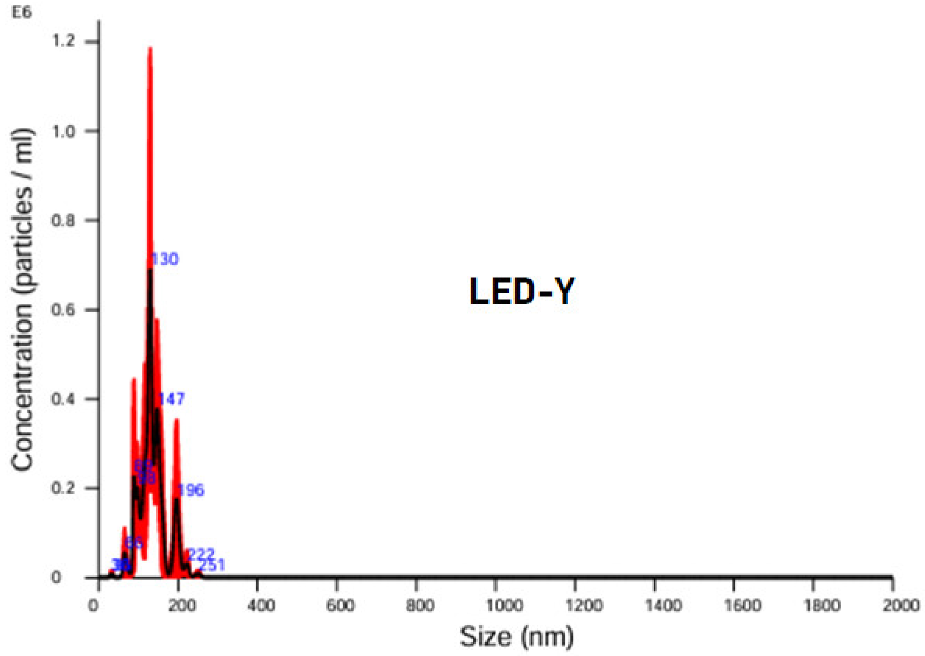
E6
Concentration (particles / ml)
LED-Y
130
147
196
222
251
Size (nm)

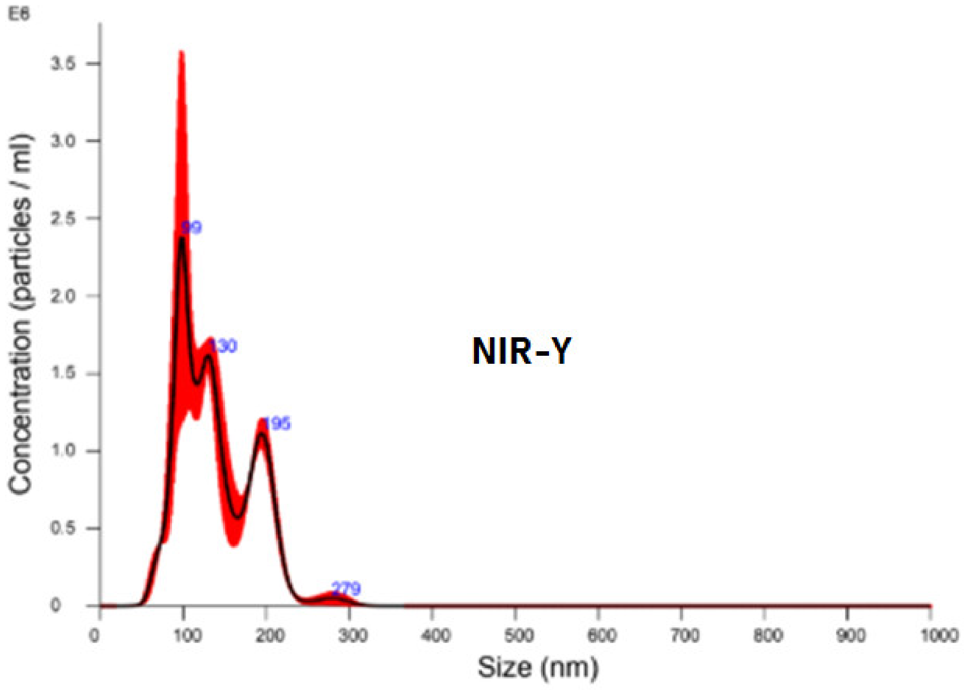
E6
Concentration (particles / ml)
NIR-Y
99
130
195
279
Size (nm)

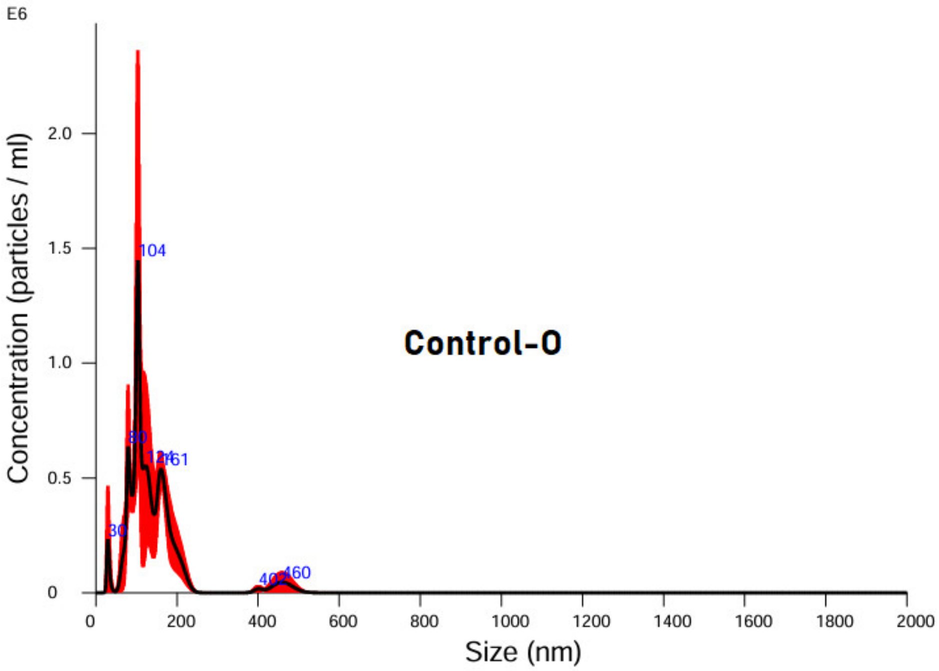
E6
Concentration (particles / ml)
2.0
1.5
1.0
0.5
0
104
80
161
30
460
Control-O
0
200
400
600
800
1000
1200
1400
1600
1800
2000
Size (nm)

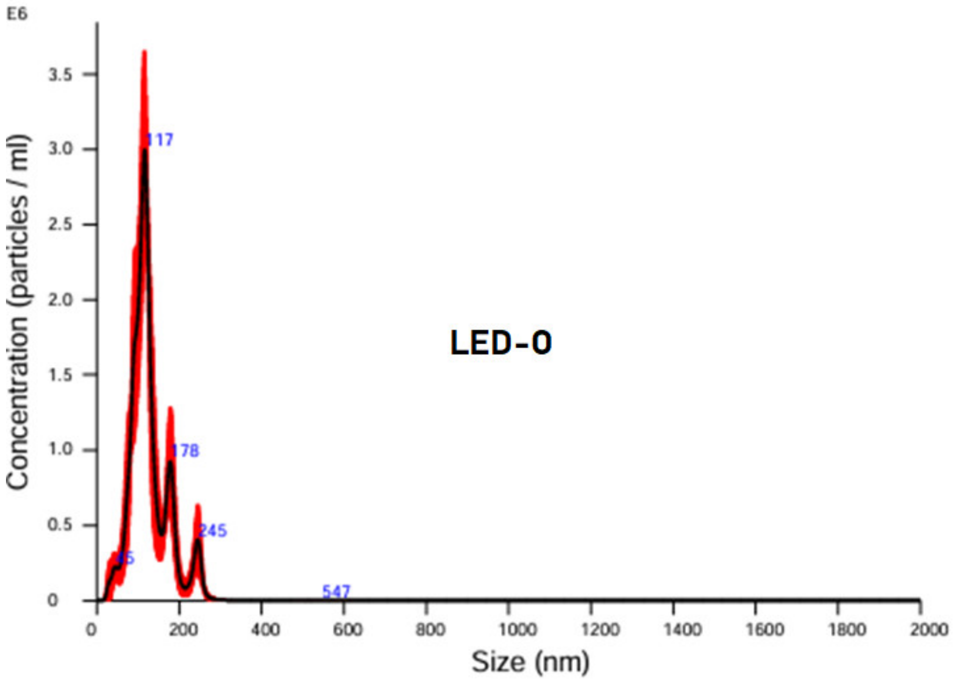
E6
Concentration (particles / ml)
3.5
3.0
2.5
2.0
1.5
1.0
0.5
0
117
178
245
547
LED-O
0
200
400
600
800
1000
1200
1400
1600
1800
2000
Size (nm)

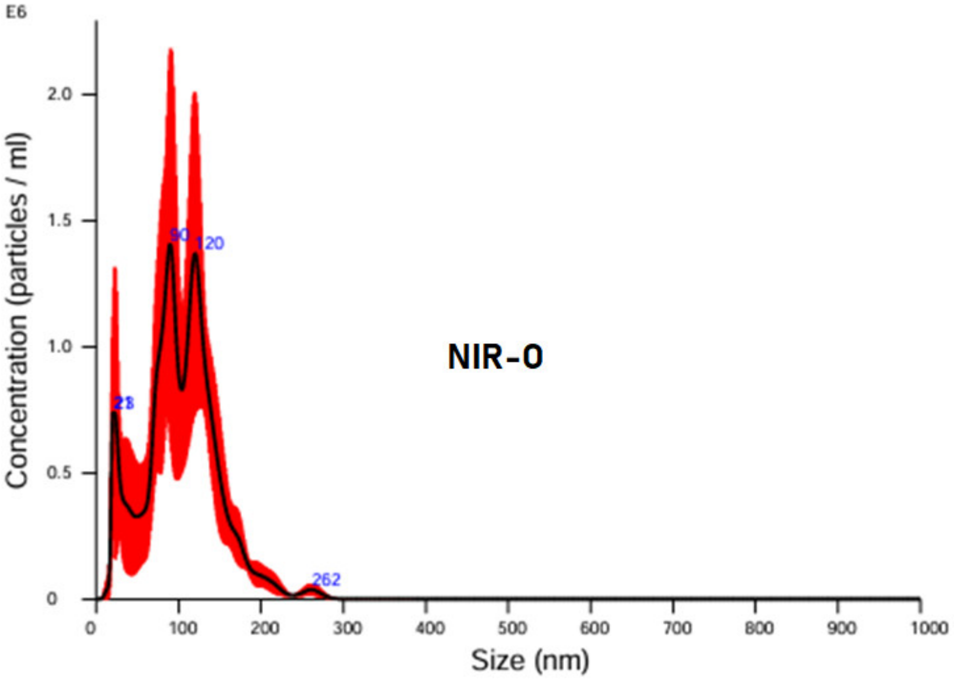
E6
Concentration (particles / ml)
2.0
1.5
1.0
0.5
0
90
120
262
NIR-O
0
100
200
300
400
500
600
700
800
900
1000
Size (nm)

Supplementary Figure S1. Nanoparticle tracking analysis (NTA) of extracellular vesicles (EVs) released from C2C12 myoblasts following photobiomodulation. Particle size distribution profiles obtained by nanoparticle tracking analysis (NTA) for extracellular vesicles (EVs) isolated from **young (Y; passage ≤5)** and **old (O; passage ≥30)** C2C12 cells under control conditions or after photobiomodulation (PBM) using either a **660 nm LED** or an **830 nm near-infrared (NIR) laser** at 5 J/cm². Each panel shows the concentration of detected particles (particles/mL) as a function of particle diameter (nm). Red curves represent the measured particle distribution, and black lines represent the smoothed distribution profile. The major particle populations detected across conditions fall within the **~50–200 nm size range**, consistent with the expected size distribution of small extracellular vesicles. Panels correspond to the following experimental conditions: EVs by untreated Young Cells (Control-Y); EVs produced by Young cells after PBM with LED (LED-Y); EVs produced by Young cells after PBM with NIR (NIR-Y); EVs by untreated old cells (Control-O); EVs produced by old cells after LED treatment, and EVs produced by old cells after NIR treatment (NIR-O).

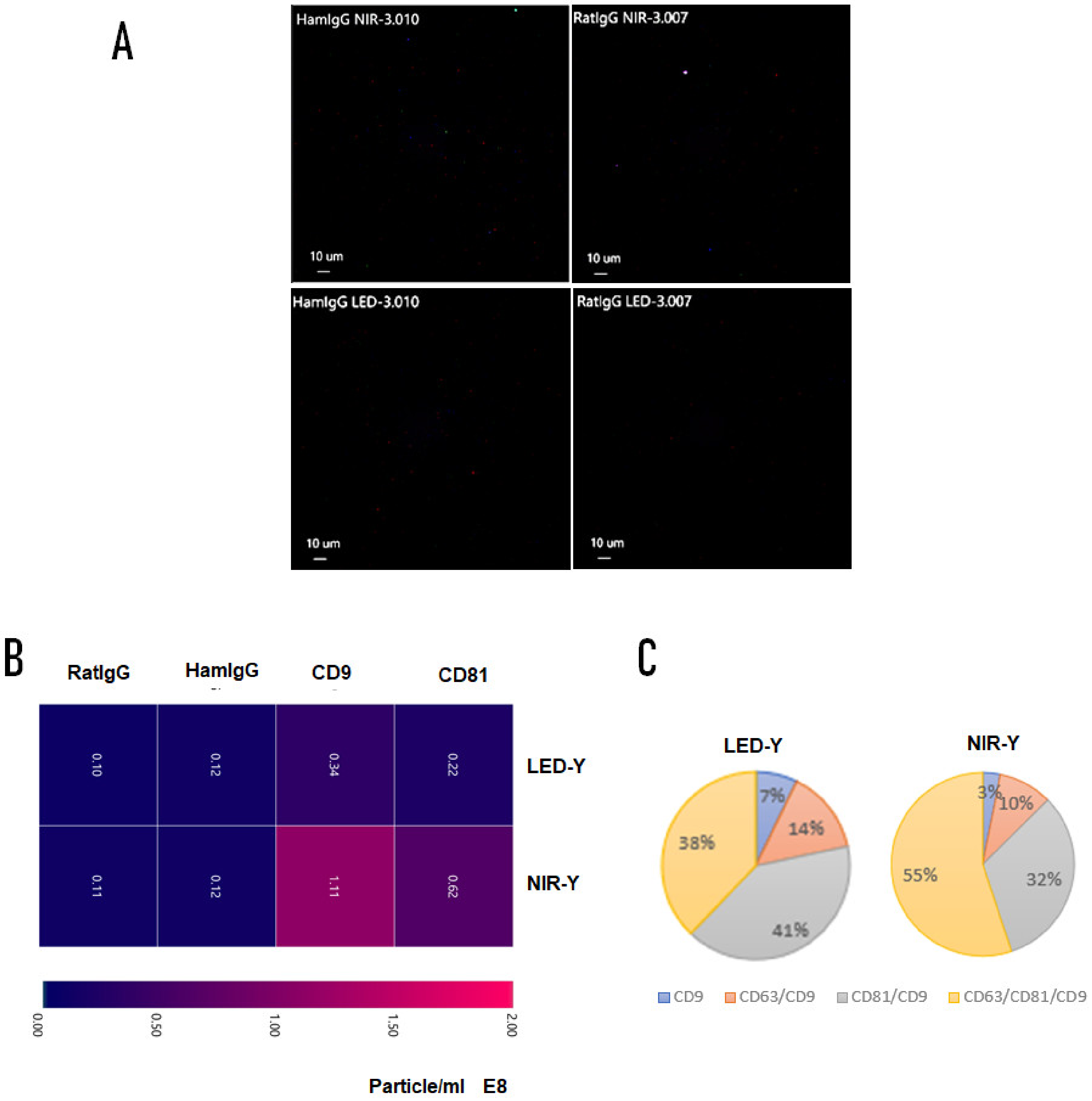

Supplementary Figure S2. Validation of ExoView detection and distribution of EV tetraspanin subpopulations in young C2C12 cells following photobiomodulation. EVs were isolated from young (≤5 passages) C2C12 cells after irradiation with 660 nm LED or 830 nm NIR at 5 J/cm² and analyzed using the ExoView platform. (A) Representative

fluorescence micrographs of negative control capture spots using hamster IgG (HamIgG) and rat IgG (RatIgG) antibodies for both LED- and NIR-treated samples, showing negligible signal and confirming assay specificity. (B) Heatmap summarizing relative abundance of EVs captured by control antibodies and canonical tetraspanin markers (CD9 and CD81) in LED- and NIR-treated samples (particles/mL $\times 10^8$). (C) Proportional distribution of EV subtypes in young cells based on ExoView colocalization analysis, including CD9 single-positive, CD63/CD9, CD81/CD9, and triple-positive CD63/CD81/CD9 vesicles.

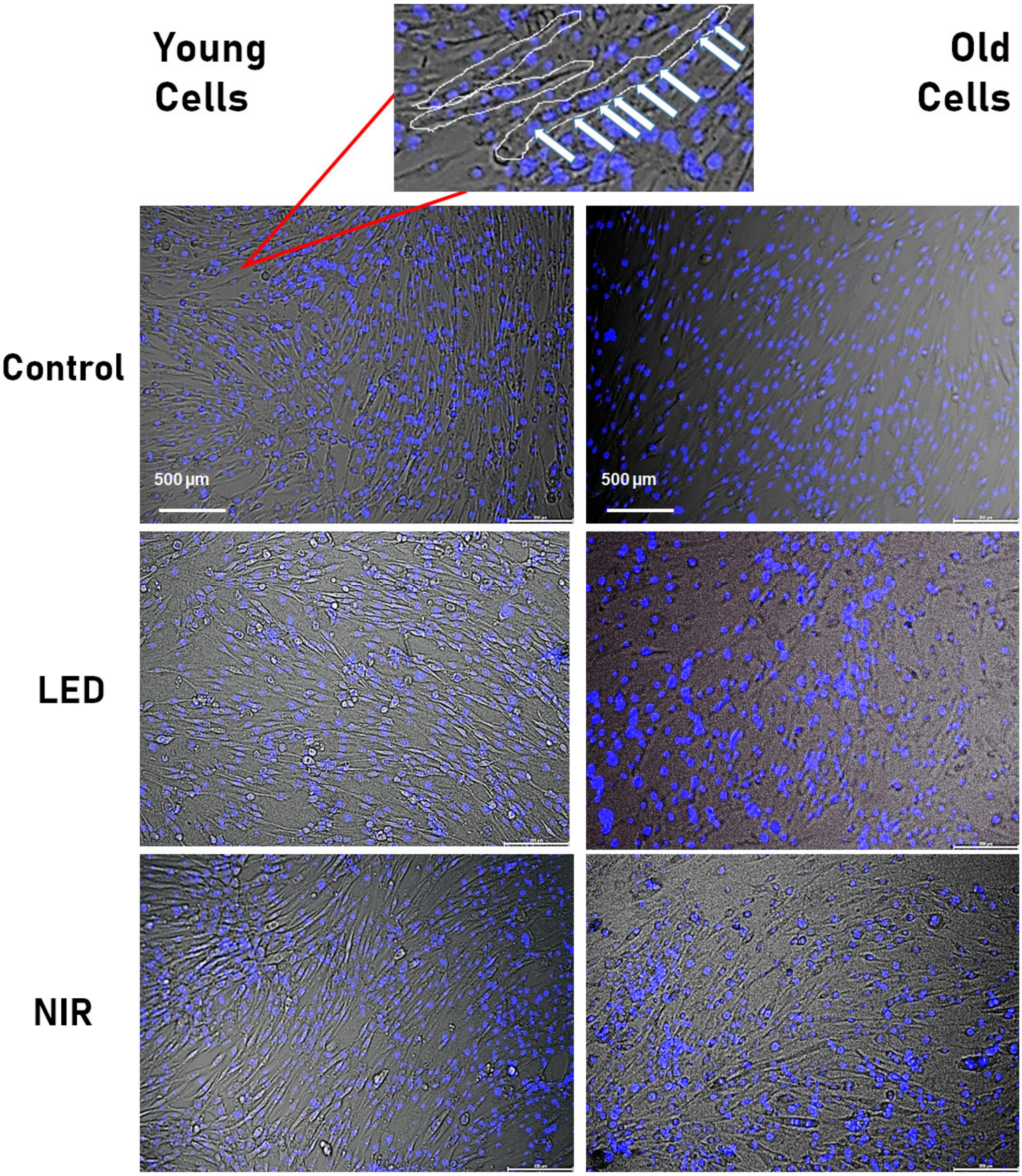

Supplementary Figure S3. Photobiomodulation effects on C2C12 myogenic differentiation. Representative bright-field images of differentiated C2C12 cultures with nuclei stained using Hoechst 33342 (blue) showing myotube formation in **young (≤5 passages)** and **old (≥30 passages)** cells under **control, 660 nm LED, and 830 nm NIR** conditions. Images illustrate the incorporation of nuclei into elongated multinucleated myotubes during differentiation. **Inset:** magnified example highlighting a representative myotube

(outlined in white) with **arrows indicating multiple nuclei incorporated within the same myofiber**, illustrating multinucleated fiber formation used for fusion index quantification. **Scale bars: 500 µm.**

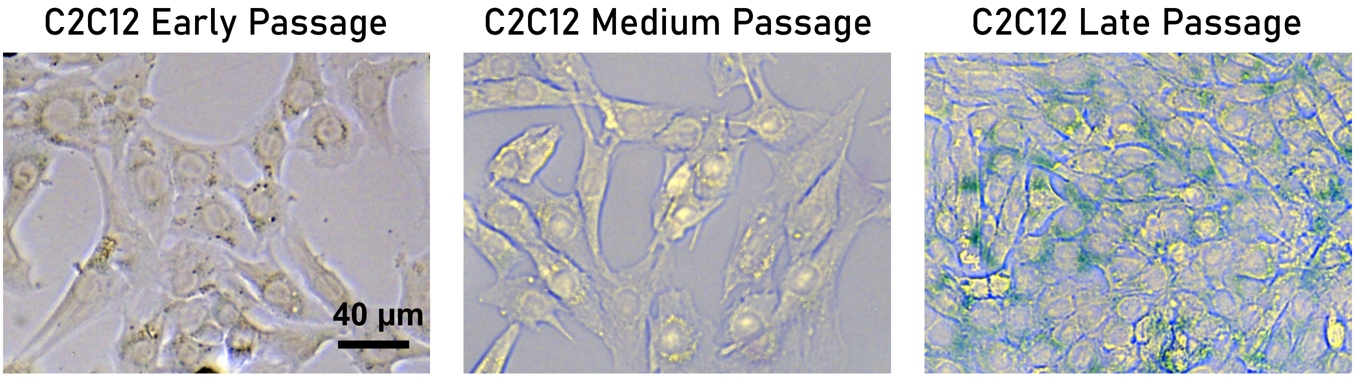

Supplementary Figure S4. Senescence-associated β-galactosidase staining in C2C12 cells across replicative passages. Representative bright-field images showing senescence-associated β-galactosidase (SA-β-gal) staining in C2C12 myoblasts at early (≤5 passages), intermediate (~P25), and late (≥30 passages) replicative stages. Cells were stained using an X-gal–based assay and imaged under identical conditions. Blue staining, indicative of SA-β-gal activity and cellular senescence, is predominantly observed in late-passage cells, while early- and intermediate-passage cultures show minimal staining. Scale bar: 40 µm. Images are representative of three independent experiments.

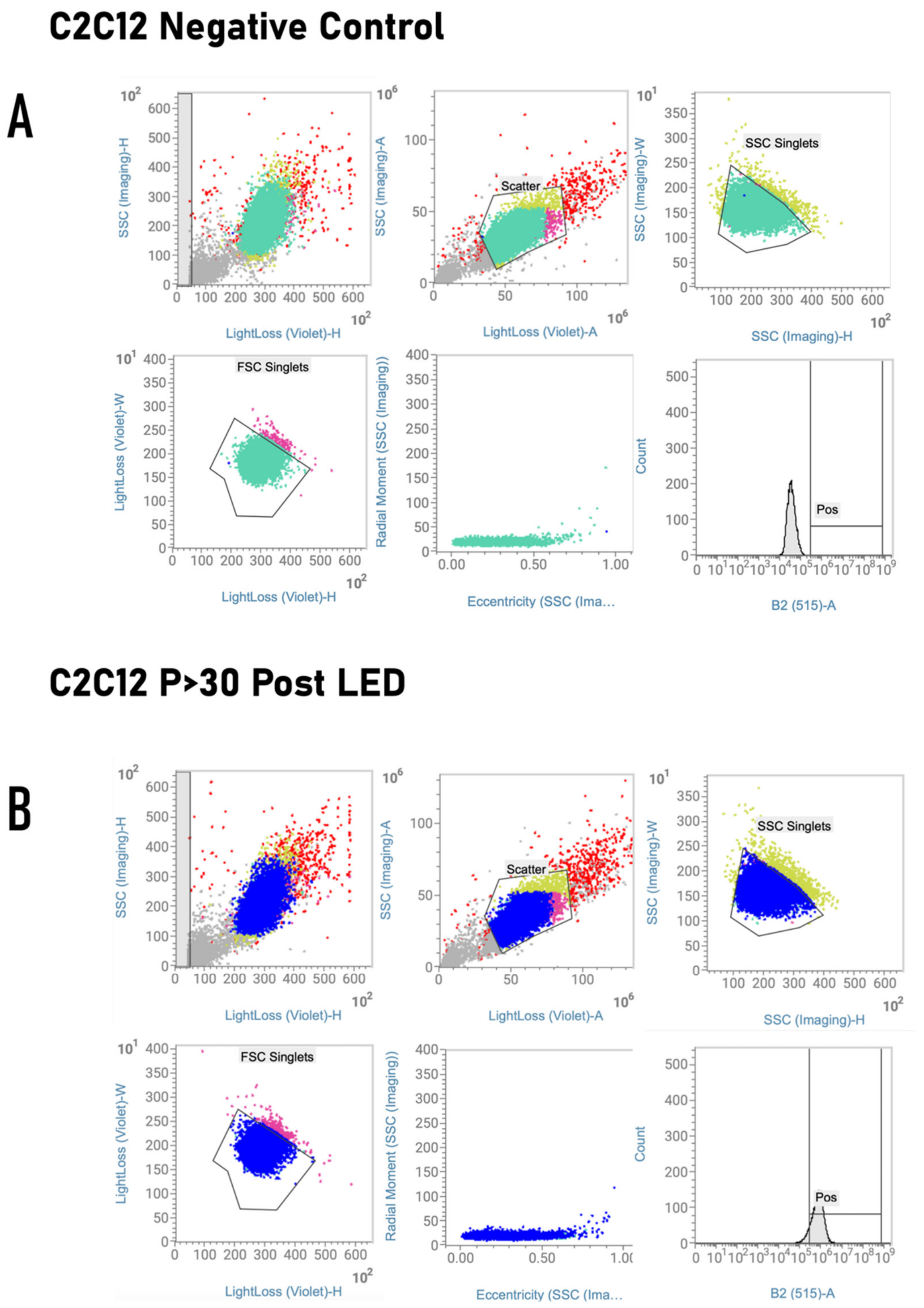

Supplementary Figure S5. Flow cytometry gating strategy for bulk ROS detection in C2C12 cells. Representative plots illustrate the sequential gating applied to define the ROS-positive population. (A) Example of gating in C2C12 control cells: debris was excluded by scatter properties, singlets identified, and fluorescence histograms gated for ROS positivity. (B) Example of gating in old passage cells after treatment with LED with the same sequential filters. ROS signal was quantified in the FITC channel (B2 [515]-A), with positive gates defined using buffer-only negative controls and validated with ROS-inducer positive controls. This gating strategy was applied consistently across all experimental groups (control, LED 660 nm, and NIR 830 nm).

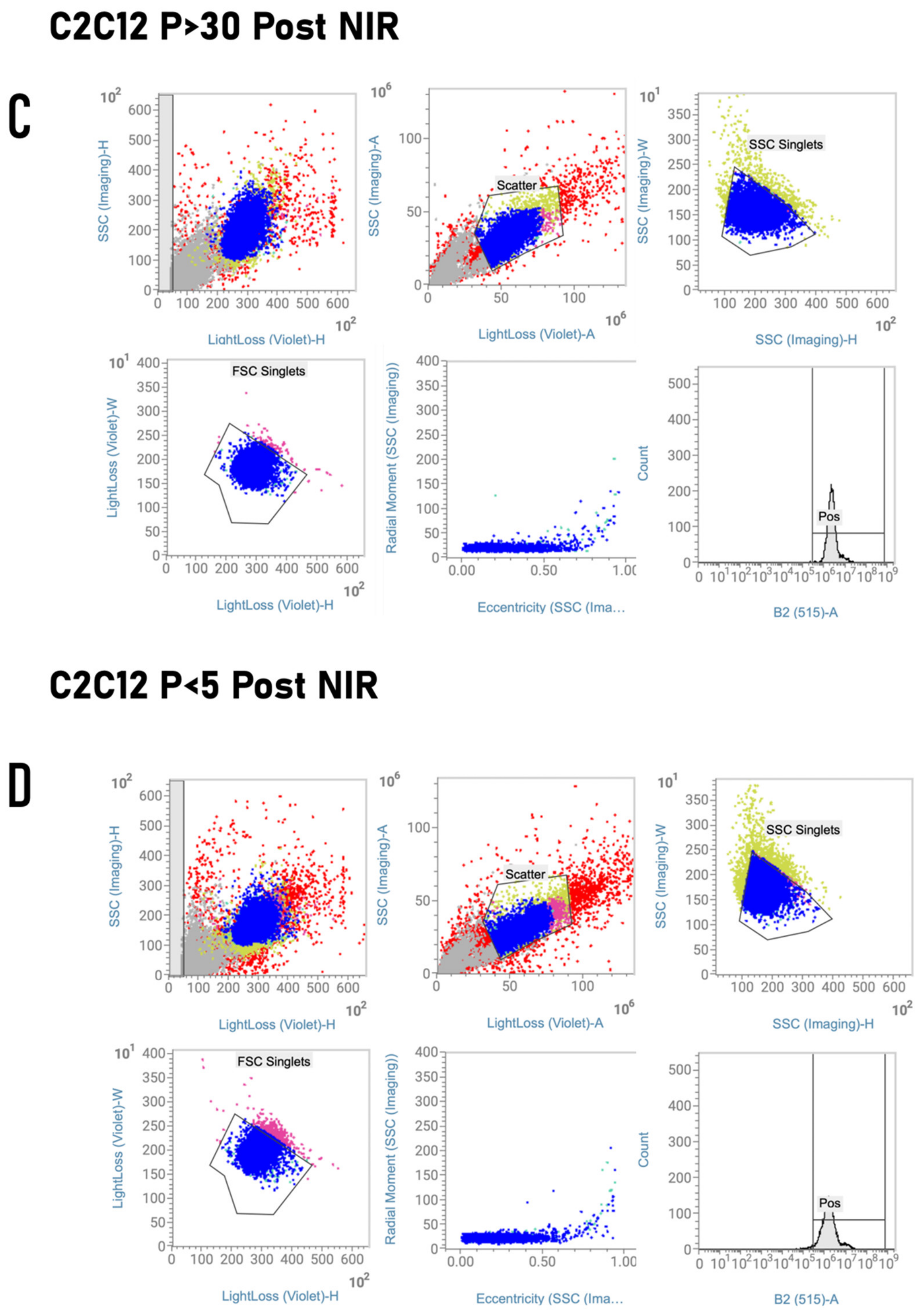

Supplementary Figure S6. Flow cytometry gating strategy for bulk ROS quantification in early- and late-passage C2C12 myoblasts after Near Infra Red (NIR) photobiomodulation (PBM). Representative plots illustrate the sequential gating workflow used to define ROS-positive cell populations across replicative stages. Debris was excluded based on light scatter properties, followed by discrimination of singlets using forward- and side-scatter parameters and imaging-based shape descriptors. Intracellular ROS levels were quantified in the FITC channel (B2 [515]-A), with positivity thresholds established using buffer-only negative controls and validated with ROS-inducer–treated positive controls. (C) Representative gating in late-passage C2C12 cells following NIR (830 nm) irradiation. (D) Representative gating in early-passage C2C12 cells following NIR (830 nm) irradiation. Identical gating criteria were applied across all experimental conditions, including control, LED (660 nm), and NIR (830 nm), to ensure consistent ROS quantification across age groups.

Supplementary Table 1. Summary of PBM experimental design and assay conditions.
Summary of photobiomodulation experimental parameters including biological variable, light source, cell passage status, irradiation dose, and culture format used across all assays.

Biological Variable / Assay	Wavelength / Source	Cell Passage /Age	PBM Dose (J/cm²)	Culture Format
**Mitochondrial membrane potential (Rh123)**	660 nm LED	Mid-Passage	0, 2.5, 5, 10	Single-cell microchamber
**Mitochondrial membrane potential (Rh123)**	830 nm NIR laser	Mid-Passage	0, 2.5, 5, 10	Single-cell microchamber
**ROS production (H2DCFDA)**	660 nm LED	Mid-Passage	0, 2.5, 5, 10	Single-cell microchamber
**ROS production (H2DCFDA)**	830 nm NIR laser	Mid-Passage	0, 2.5, 5, 10	Single-cell microchamber
**Cell viability (CCK-8)**	660 nm LED	Early / Late Passage	0, 2.5, 5, 10, 15	96-well plate
**Cell viability (CCK-8)**	830 nm NIR laser	Early / Late Passage	0, 2.5, 5, 10, 15	96-well plate
**ATP production**	660 nm LED	Early / Late Passage	5	96-well plate
**ATP production**	830 nm NIR laser	Early / Late Passage	5	96-well plate
**ROS production (Flow-cytometry)**	660 nm LED	Early / Late Passage	5	24-well plate
**ROS production (Flow-cytometry)**	830 nm NIR laser	Early / Late Passage	5	24-well plate
**Extracellular vesicle release (NTA)**	660 nm LED	Early / Late Passage	5	24-well plate
**Extracellular vesicle release (NTA)**	830 nm NIR laser	Early / Late Passage	5	24-well plate
**Extracellular vesicle Characterization (ExoView)**	660 nm LED	Early Passage	5	24-well plate
**Extracellular vesicle Characterization (ExoView)**	830 nm NIR laser	Early Passage	5	24-well plate
**Cell migration (culture-insert)**	660 nm LED	Early / Late Passage	5	24-well plate
**Cell migration (culture-insert)**	830 nm NIR laser	Early / Late Passage	5	24-well plate
**Myogenic differentiation / fusion index**	660 nm LED	Early / Late Passage	5	24-well plate
**Myogenic differentiation / fusion index**	830 nm NIR laser	Early / Late Passage	5	24-well plate

Supplementary Table 2. Summary of Single-Cell PBM Responses and Dose Selection (C2C12, Passage 25). Purpose: Acute mitochondrial and redox sensing to identify optimal fluence.

Output (Single-cell)	LED (660 nm)	NIR (830 nm)	Dose Dependence / Interpretation	
**Mitochondrial membrane potential (ΔΨm, Rh123)**	↑ at 5 J/cm²	↑ at 5 J/cm²	Plateau ≥10 J/cm²	Both sources enhance mitochondrial polarization; optimal response at 5 J/cm²
**Intracellular ROS (H_2DCFDA)**	↑ at 5 J/cm²	↑ at 5 J/cm²	Biphasic	Moderate ROS increase consistent with mitohormetic activation
**Source comparison**	Comparable Responses	Comparable Responses	No strong wavelength-specific differences at single-cell level	
**Dose selection outcome**	5 J/cm² selected	5 J/cm² selected	Guided dose for population-level assays	

↑ increase relative to baseline

Supplementary Table 3. Summary of Population-Level and Functional PBM Effects by Age and Light Source. Purpose: Biological outcomes under optimal fluence (5 J/cm²).

Output / Assay	Young Cells LED (660 nm)	Young Cells NIR (830 nm)	Old Cells LED (660 nm)	Old Cells NIR (830 nm)	Key Biological Trend
**Metabolic activity (CCK-8)**	↑↑	↑	↑↑	↑	LED produces stronger metabolic activation
**ATP production**	↑ (≈ NIR)	↑ (≈ LED)	↑↑ (LED > NIR)	↑ (< LED)	Aged cells show greater ATP response to LED
**Bulk ROS (% positive cells)**	↑↑	↑↑	↑↑	↑↑	Strong redox activation independent of irradiation modality
**EV particle release (NTA)** [Phenotype in early-passage]	↑ [↑ CD9/CD81 EVs]	↑↑ [↑↑ triple-positive EVs]	↑↑	↑	Source sensitivity shifts with age [NIR favors canonical small EV populations]
**Migration (wound healing)**	↑↑ sustained (24–36 h)	↑ transient / delayed	↑ early only (transient)	No sustained effect	Migration weak and strongly age-dependent; LED modestly improves young cell closure
**Myogenic fusion (fusion index)**	Trend ↑	Significant ↑↑	NS	Significant ↑↑	NIR strongly promotes differentiation

↑ modest increase; ↑↑ strong increase; NS not significant.
EV source sensitivity shifts with age; NIR favors canonical small EV populations

Supplementary Methods S1. Quantification of wound closure dynamics

The wound area was measured using ImageJ software (NIH, USA), and values were normalized to the initial wound size at 0 h [63].

Relative gap (%):

The remaining wound area at time *t* expressed as a percentage of the baseline gap:

$$\text{Relative gap (\%)} = \frac{W_t}{W_0} \times 100 \quad (1)$$

Where W_0 is the wound gap at 0 h and W_t is the gap at time t.

Relative closure rate (%/h):

The average rate of closure from baseline to a given time point, expressed as percent reduction in relative gap per hour:

$$\text{Relative closure rate (\%/h)} = \frac{100 - \text{Relative gap at time } t}{\Delta t} \quad (2)$$

Where Δt is the elapsed time (h) since 0 h.

Interval-specific closure rate (%/h):

The rate of closure calculated between two consecutive time points, reflecting dynamic changes across different phases of migration:

$$\text{Interval closure rate (\%/h)} = \frac{\text{Relative gap at start} - \text{Relative gap at end}}{\Delta t} \quad (3)$$

where “start” and “end” represent two successive measurement times.

All experiments were performed in triplicate, and results were expressed as mean ± SEM.
